# Synergistic antibacterial effects of postbiotics combined with linezolid and amikacin against nosocomial pathogens

**DOI:** 10.3389/fcimb.2025.1616501

**Published:** 2025-08-14

**Authors:** Elif Yaprak Çolak, Nizami Duran

**Affiliations:** Department of Medical Microbiology, Medical Faculty, Hatay Mustafa Kemal University, Antakya-Hatay, Türkiye

**Keywords:** postbiotics, linezolid, amikacin, nosocomial infections, antimicrobial synergy, microbiome therapy

## Abstract

**Background and Aim:**

The global rise in antimicrobial resistance (AMR) has rendered many conventional antibiotics less effective, particularly against nosocomial pathogens such as *Staphylococcus aureus*, *Escherichia coli*, *Pseudomonas aeruginosa*, and *Proteus mirabilis*. This study investigated the antimicrobial and synergistic effects of postbiotics derived from *Lacticaseibacillus casei*, *Lactobacillus bulgaricus*, *Enterococcus faecium*, and *Streptococcus thermophilus*, administered alone or in combination with either linezolid (for *S. aureus*) or amikacin (for Gram-negative strains).

**Materials and methods:**

Postbiotics were obtained through anaerobic fermentation, followed by centrifugation and filtration. Cytotoxicity was assessed via MTT assays on Vero cell lines. Infection models involving pathogen-specific adhesion and invasion assays were used, with CFU/mL quantification and statistical evaluation by one-way ANOVA and Tukey’s *post hoc* test.

**Results:**

The postbiotics exhibited potent antimicrobial activity across all tested pathogens. Combined with linezolid, the dual and triple postbiotic formulations significantly enhanced antibacterial effects against *S. aureus* from the early hours of incubation. Similarly, combinations with amikacin produced potent synergistic effects against *E. coli*, *P. aeruginosa*, and *P. mirabilis*, particularly in triple combinations involving *L. casei* and *L. bulgaricus*. Postbiotics sometimes outperformed antibiotics, such as ST+LC postbiotics against *P. mirabilis*. These findings suggest that postbiotics can enhance antibiotic efficacy-possibly by modulating membrane permeability, disrupting biofilms, or altering bacterial communication systems. Their low cytotoxicity and pathogen-specific responses indicate that postbiotics are safe and may be tailored for targeted use.

**Conclusions:**

In conclusion, postbiotic-antibiotic combinations, especially with linezolid and amikacin, present promising low-toxicity, synergistic therapeutic strategies. These results lay a strong foundation for advancing microbiome-based adjunct therapies to combat AMR in clinical settings.

## Introduction

Antimicrobial resistance (AMR) has become one of the most serious global health threats in modern medicine. Due to the increasing resistance of microorganisms, the effectiveness of antibiotics in treating infectious diseases worldwide has gradually decreased. Nosocomial pathogens, particularly *Pseudomonas aeruginosa*, *Staphylococcus aureus*, *Proteus mirabilis*, and *Escherichia coli*, can rapidly develop resistance to existing antimicrobial agents through various mechanisms of gene transfer. This complicates the treatment of infections, increasing mortality and morbidity rates (O’Neill, 2016; Murray et al., 2024).

According to the WHO (2020), antimicrobial resistance (AMR) causes approximately 700.000 deaths annually worldwide, a number that could rise to 10 million by 2050 if no effective interventions are made. Particularly, multidrug-resistant (MDR) pathogens such as *Escherichia coli*, *Pseudomonas aeruginosa*, and *Proteus mirabilis* are responsible for a large proportion of nosocomial infections and exhibit increasing resistance to conventional antibiotics (Cassini et al., 2019; World Health Organization, 2019).

The limited effectiveness of conventional antibiotics and the slow development of new antibiotics have increased the interest in alternative infection control strategies. In this context, the discovery and use of bioactive compounds of natural origin have gained significant importance. Probiotics are one of the most remarkable areas of research in this field, supporting gastrointestinal system health and exhibiting antimicrobial effects (Aguilar-Toalá et al., 2018; Asadi et al., 2024). Probiotic microorganisms can inhibit the proliferation of pathogens by creating an acidic microenvironment through organic acid production, while also enhancing the host immune response by increasing secretory IgA and serum IgA levels. In addition, non-specific immune responses are triggered by stimulating phagocytosis, increasing natural killer cell activity, and supporting cell-mediated immunity (Hill et al., 2014; Binda et al., 2020).

In recent years, postbiotics metabolic byproducts produced by probiotics have garnered increasing interest in the scientific community. Postbiotics contain short-chain fatty acids, enzymes, vitamins, antimicrobial peptides, and other bioactive components, exhibiting anti-inflammatory, immunomodulatory, and antimicrobial effects (Da et al., 2025; Kumaari and Mohanasrinivasan, 2025). These biological activities have brought the evaluation of postbiotics as potential adjuvant agents in the management of infectious diseases to the agenda.

According to the latest scientific consensus, the term “postbiotics” broadly refers to non-viable microbial products or metabolic byproducts with biological activity, including SCFAs, lipids, proteins, peptides, and enzymes (Salminen et al., 2021). While the composition of postbiotics can be diverse, research may focus on specific fractions depending on the analytical approach and study aim. In this study, although the term “postbiotics” is retained to describe the overall biological nature of the preparations, only the protein-based content was quantified using the Bradford assay (Kruger, 2009). This has been clearly stated in the methodology to ensure clarity and transparency.

Although the antimicrobial properties of postbiotics have been demonstrated in various studies, the therapeutic synergy these components can create when used in conjunction with conventional antibiotics has not yet been sufficiently investigated. The potential of postbiotics to enhance the efficacy or mitigate the toxicity of antibiotics, particularly against pathogens that exhibit multidrug resistance, is quite promising. Understanding these synergistic interactions may contribute to the development of low-toxicity, effective combination therapies in the fight against AMR (Ribeiro et al., 2024; Kumaari and Mohanasrinivasan, 2025; Pérez-López et al., 2025).

Therefore, it is essential to systematically evaluate the potential synergistic effects that may occur when postbiotics are combined with antibiotics. This study aims to shed light on new therapeutic strategies in the fight against antimicrobial resistance by examining the potential interactions between postbiotics and antibiotics.

In this study, the postbiotics (bioactive metabolic products) of probiotic microorganisms, including *Lactobacillus bulgaricus*, *Lacticaseibacillus casei*, *Enterococcus faecium*, and *Streptococcus thermophilus*, were evaluated for their antimicrobial activity against major human pathogens: *P. aeruginosa*, *S. aureus*, *P. mirabilis*, and *E. coli*.

The investigation focused on three main objectives: (i) to assess the antimicrobial properties of individual postbiotic compounds, (ii) to explore the synergistic effects of combined postbiotics, and (iii) to evaluate the interactions of these postbiotic mixtures with conventional antibiotics, specifically ampicillin and amikacin.

The study aimed to highlight the potential of these probiotic-derived bioactive substances as alternative therapeutic strategies against antibiotic-resistant pathogens. A more comprehensive understanding of the antimicrobial roles of such metabolites could significantly contribute to the development of novel biologically based approaches for combating antimicrobial resistance.

## Materials and methods

This study investigated the antimicrobial and synergistic effects of postbiotics derived from*Lacticaseibacillus casei*, *Lactobacillus bulgaricus*, *Enterococcus faecium*, and *Streptococcus thermophilus* against *Staphylococcus aureus*, *Escherichia coli*, *Pseudomonas aeruginosa*, and *Proteus mirabilis* (**Supplementary Figure S1**).

### Bacterial strains

The probiotic and pathogenic bacterial strains used in this study *Lacticaseibacillus casei* (ATCC 393), *Streptococcus thermophilus* (ATCC 19258), *Lactobacillusbulgaricus* (ATCC 11842), *Enterococcus faecium* (Fisher Scientific, Cat. No. 50-238-04082), *Staphylococcus aureus* (ATCC 43300), *Pseudomonas aeruginosa* (ATCC BAA-2108), *Escherichia coli* (ATCC BAA-196), and *Proteus mirabilis* (ATCC 7002) were commercially obtained from the Microbiology Culture Collection of the Refik Saydam National Public Health Institute (Ankara, Türkiye) and the American Type Culture Collection (ATCC, USA).

*L.casei*, *L.bulgaricus*, and *S.thermophilus* were grown on de Man, Rogosa, and Sharpe (MRS) agar (Merck, Germany) in an anaerobic chamber incubated at 37°C (Patel et al., 2023). Then, the supernatant was collected by centrifugation at 6000 rpm (revolutions per minute) for 30 minutes at 4°C and filtered through a 0.45 μm (micron) filter (Aguilar-Toalá et al., 2021a). The blank medium MRS was incubated for 48 hours under the same conditions, centrifuged at 6000 rpm for 30 minutes at 4°C, and filtered through a 0.45-μm filter as a control.

Postbiotic supernatants were quantified based on total protein content using the Bradford Protein Assay (Bio-Rad, USA), following the manufacturer’s protocol. Absorbance was measured at 595 nm using a microplate reader (Bradford, 1976).

This method selectively measures soluble proteins and peptides. Therefore, the quantitative biochemical evaluation in this study was limited to the protein-based fraction of the postbiotic preparations and did not include non-protein components such as SCFAs or lipids (Kruger, 2009).

### Antibiotics

Linezolid and amikacin were selected as the standard drugs for these experiments and were commercially obtained (Sigma-Aldrich, USA).

### Cell culture

The Vero cell line (African Green Monkey Kidney Cells, ATCC CCL-81) was used in the study. Non-toxic concentrations of postbiotics were determined in the Vero cell line (Park et al., 2023). RPMI-1640 containing 10% fetal calf serum (FBS), 10 mM HEPES, 100 IU/ml penicillin/streptomycin, and four mM glutamine was used as a cell culture medium. Cell cultures were cultivated in a humidified incubator at 37°C and 5% CO_2_. Cell density was adjusted to 1 × 10^6 cells/mL for proliferation and activity experiments.

Bacteria [1×10^8^ CFU (Colony Forming Unit) ml¹] were added to Vero cells at 100 MOI for 6 hours at 37°C in a humidified atmosphere supplemented with 5% CO_2_ for bacterial adhesion and invasion (Elhadidy et al., 2024). Cell incubation was continued for 96 hours.

### Cytotoxicity tests

The Vero cell line was used in cytotoxicity studies. First, the non-toxic concentrations of these compounds were determined (Matsuoka et al., 2020). Activity assays were performed in 96-well flat-bottomed microplates. Cells were inoculated into the wells with RPMI 1640 medium containing 10% fetal calf serum at a concentration of 1 × 10^6 cells/mL. The non-toxic concentrations of the postbiotics from *S. thermophilus* and *L. casei*, as well as antibiotics, were determined in Vero cell cultures using the MTT method (Mosmann, 1983). The cytotoxicity of postbiotics was assessed on Vero cells using the MTT assay, and non-toxic concentrations were determined based on a viability threshold of greater than 80% as reported by Park et al (Park et al., 2023).

### MTT (3-(4,5-dimethylthiazol-2-yl)-2,5-diphenyltetrazolium bromide) assay

The MTT assay, originally described by Mosmann, is widely utilized to evaluate cell viability and cytotoxicity. MTT is taken up by metabolically active cells and reduced to an insoluble purple formazan by mitochondrial dehydrogenase enzymes. The intensity of the resulting color is directly proportional to the number of viable cells (Mosmann, 1983).

This method evaluated the cytotoxic effects of postbiotics and antibiotics (ampicillin and amikacin) on Vero cells. Twenty-four hours prior, cells were seeded into 96-well plates (1 × 10^5^ cells/well) in 100 µL RPMI-1640 and incubated for 24 h at 37°C with 5% CO_2_ to promote adherence. Serial dilutions of postbiotics (20.0-2.5 µg/mL) and antibiotics (0.25-2.0 µg/mL) were then applied.

After incubation, 100 µL of MTT solution was added and left for 2 hours. The reaction was stopped with 100 µL of DMSO, and absorbance was measured at 570 nm using a microplate reader. Microsoft Excel was used to calculate cell viability (%) and determine IC_50_ values via a logarithmic slope curve. Each concentration was tested in triplicate, and dose-response relationships were established.

### Activity studies

First, non-toxic concentrations of *L. casei*, *L. bulgaricus*, and *S. thermophilus* postbiotics were determined in Vero cell cultures. Maximum non-toxic concentrations of *L. casei*, *L. bulgaricus*, and *S. thermophilus* postbiotics (12.5 µg/mL, 12.5 µg/mL, and 25 µg/mL, respectively) were selected as test concentrations in activity studies. Morphology was analyzed using an inverted microscope daily to examine the effects on Vero cell growth, and cell viability was determined (Matsuoka et al., 2020).

### Preparation of bacterial cultures and infection protocol

*Bacterial strains (S. aureus*, *E. coli*, *P. aeruginosa*, and *P. mirabilis*) were grown in the Brain Heart Infusion (BHI) medium until the exponential phase. Bacterial suspensions were collected by centrifuging at 4000 g for 20 minutes and purified by washing with PBS. The infection rate was adjusted using RPMI-1640 medium so that the MOI (Multiplicity of Infection) was 100 (i.e., the ratio of bacteria to cells) (Elhadidy et al., 2024).

### Cell infection and adhesion test

After washing the cell monolayer with RPMI-1640 medium, the prepared bacterial suspensions were added to the wells and incubated for 2 hours at 37°C in a 5% CO_2_ environment (Abed et al., 2021). During the experiment, a 1 mL sample of the bacterial suspension was incubated in parallel to check for bacterial growth or death. After the incubation period, the Vero cells were washed three times with RPMI-1640 medium to remove bacteria that had not adhered to the cell surface. The cells were then lysed with PBS solution containing 0.1% Triton X-100 for 15 minutes at 37°C. The colony counting method was used to determine the total number of adhered and internalized bacteria (Kanmani et al., 2020).

### Invasion test and internal cell bacteria count

To determine the bacteria that have invaded the cell, bacteria that adhered to the cell surface but were not internalized were killed by incubating for 1 hour with RPMI-1640 medium containing 100 µg/ml gentamicin. Then, the cells were washed three times with PBS and lysed with 0.1% Triton X-100 solution to release the internalized bacteria they contained. After the lysis process, the bacteria released were seeded into the medium by serial dilution and incubated at 37°C to perform a colony count. The number of bacteria that did not show invasion but adhered was calculated by subtracting the internalized bacteria obtained after gentamicin treatment from the total number of cell-bound bacteria (Fanning et al., 2022).

### Statistical analysis

GraphPad Prism software (version 10) was used for statistical analysis. The mean and standard deviation of at least three experiments were calculated. Comparisons between each group and between groups were performed with one-way ANOVA tests. Only findings with a p-value less than 0.05 were considered significant. Experiments with two subgroups were analyzed using a two-tailed unpaired t-test. Experiments with three or more univariate subgroups were analyzed using one-way ANOVA. *Post hoc* analysis was performed using Tukey’s multiple comparison tests between any two groups. Each statistical test is explained in the figure or table descriptions. Only data containing p<0.05 were considered statistically significant. Symbols for different test significance levels are assigned as follows: * p<0.05, ** p<0.001, *** p<0.0001, **** p<0.00001, and not significant (ns) for p>0.05.

## Results

**Figure 1:**
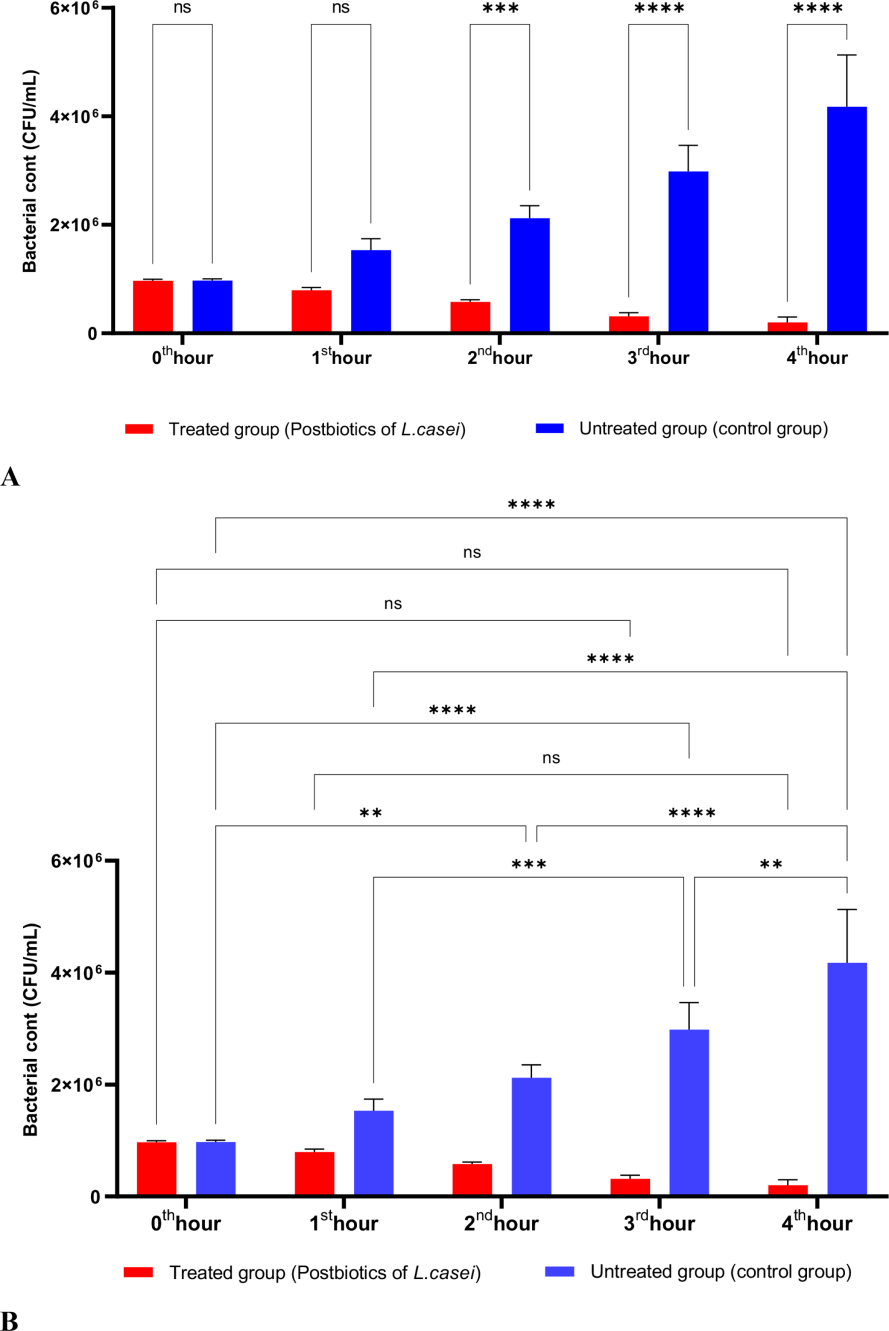
A comparative analysis of the inhibitory effects of *L. casei* postbiotics on the growth of *S. aureus*, compared to the control group **(A, B)**. **p < 0.01, ***p < 0.001, ****p < 0.0001. “ns” stands for “not significant”.

In **Figure 1**, *S. aureus* bacterial suspension treated with *L. casei* postbiotics (1 × 10^6 bacteria/mL) from the 0th hour to the 4th hour of incubation was compared with the control suspension without postbiotics. The data obtained revealed that *L. casei* postbiotics inhibited *S. aureus* growth from the first hour; this effect became statistically significant at the 2nd, 3rd, and 4th hours (**Figures 1A, B**).

**Figure 2:**
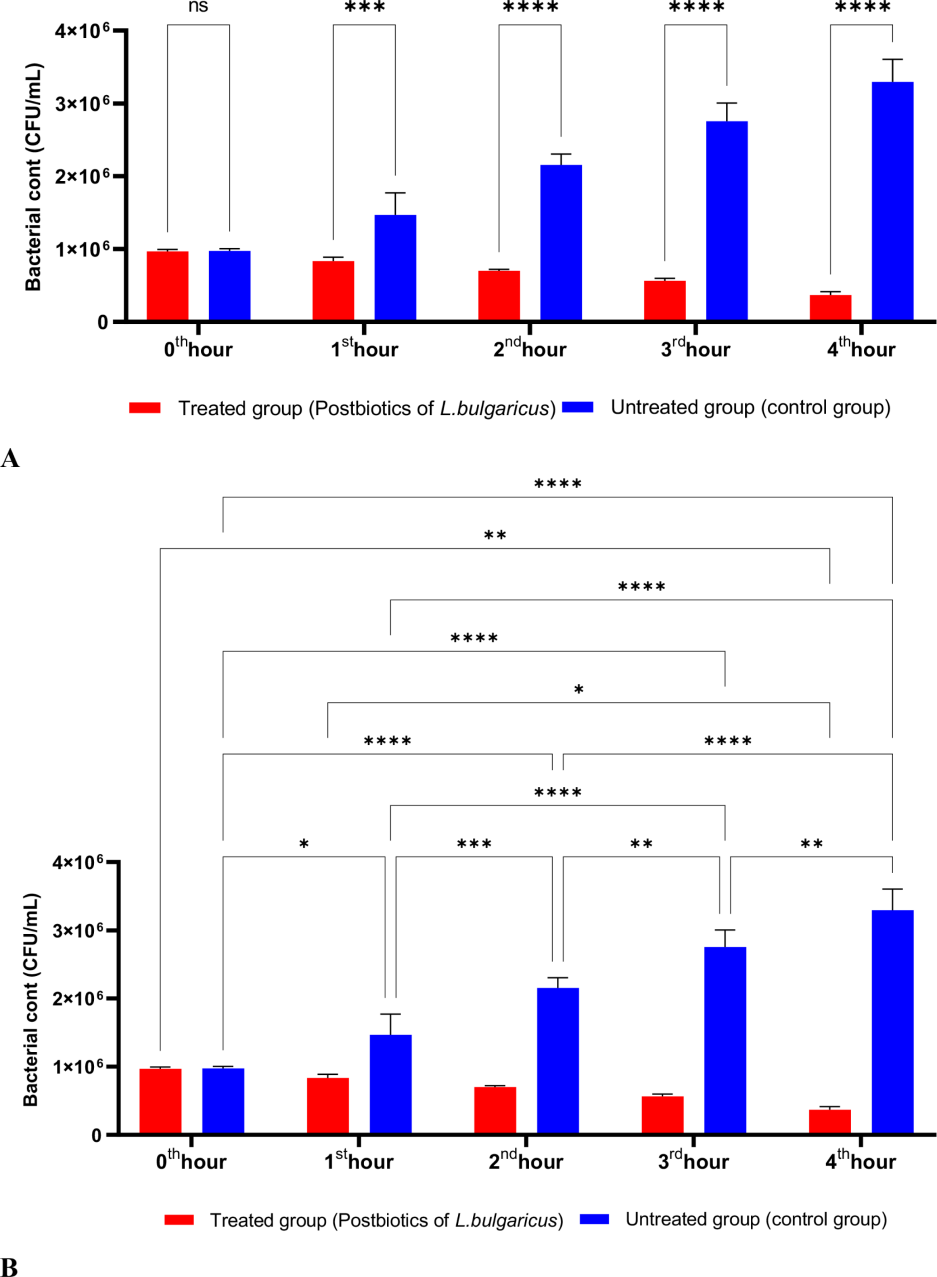
A comparative analysis of the inhibitory effects of *L. bulgaricus* postbiotics on the growth of *S. aureus*, compared to the control group **(A, B)**. *p < 0.05, **p < 0.01, ***p < 0.001, ****p < 0.0001. “ns” stands for “not significant”.

**Figure 2** shows the effects of *L. bulgaricus* postbiotics on *S. aureus*. The results indicate that *L. bulgaricus* exhibits an inhibitory mechanism similar to *L. casei* postbiotics against *S. aureus*. However, the inhibitory effect of *L. bulgaricus* postbiotics on *S. aureus* growth significantly decreased after the first hour of incubation. In contrast, the inhibition of *S. aureus* growth continued to increase notably during the second, third, and fourth hours of incubation (see **Figures 2A, B**).

**Figure 3:**
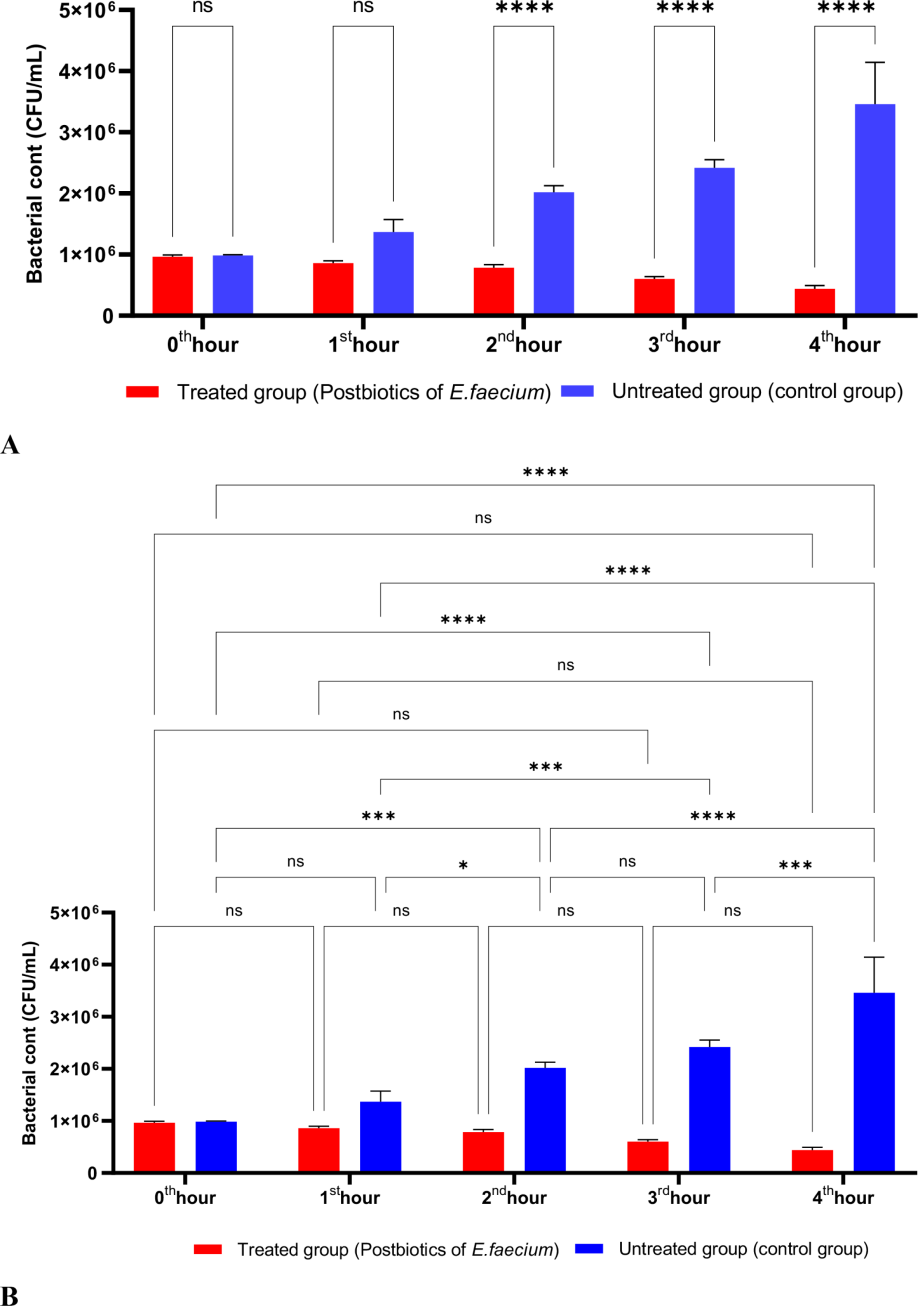
A comparative analysis of the inhibitory effects of *E. faecium* postbiotics on the growth of *S. aureus*, compared to the control group **(A, B)**. *p < 0.05, ***p < 0.001, ****p < 0.0001. “ns” stands for “not significant”.

As shown in **Figure 3**, the postbiotics of *E. faecium* had effects similar to those of *L. casei*. It was observed that postbiotics of *E. faecium* decreased *S. aureus* growth during the first hour of incubation, although this reduction was not statistically significant. However, the inhibition of bacterial growth increased significantly during the 2nd, 3rd, and 4th hours of incubation (**Figures 3A, B**).

**Figure 4:**
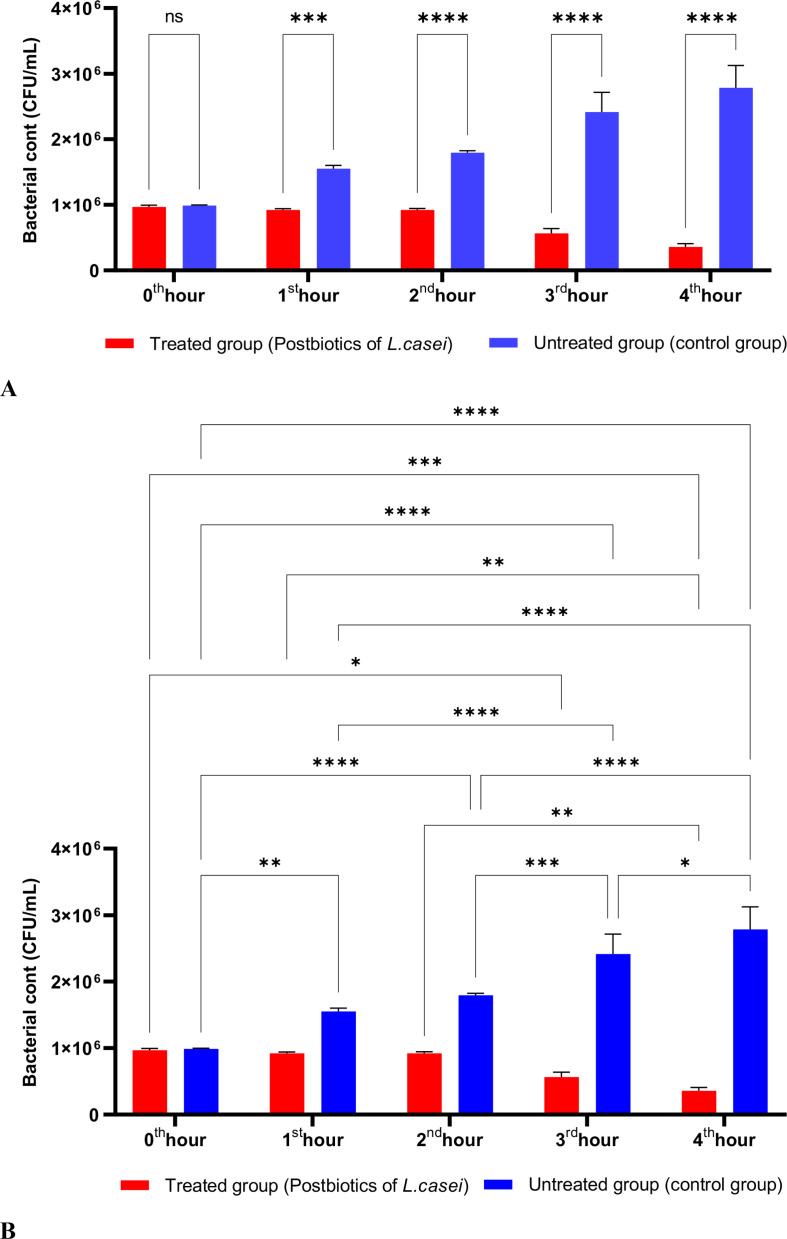
A comparative analysis of the inhibitory effects of *S. thermophilus* postbiotics on the growth of *S. aureus*, compared to the control group **(A, B)**. *p < 0.05, **p < 0.01, ***p < 0.001, ****p < 0.0001. “ns” stands for “not significant”.

The postbiotics derived from *S. thermophilus* demonstrated inhibitory activity against *S. aureus*, similar to the effects observed with postbiotics from *L. bulgaricus*. The postbiotics from *S. thermophilus* significantly inhibited the growth of *S. aureus* starting from the first hour of incubation. This inhibitory activity increased and became more pronounced at the second, third, and fourth hours of incubation (**Figures 4A, B**).

**Figure 5:**
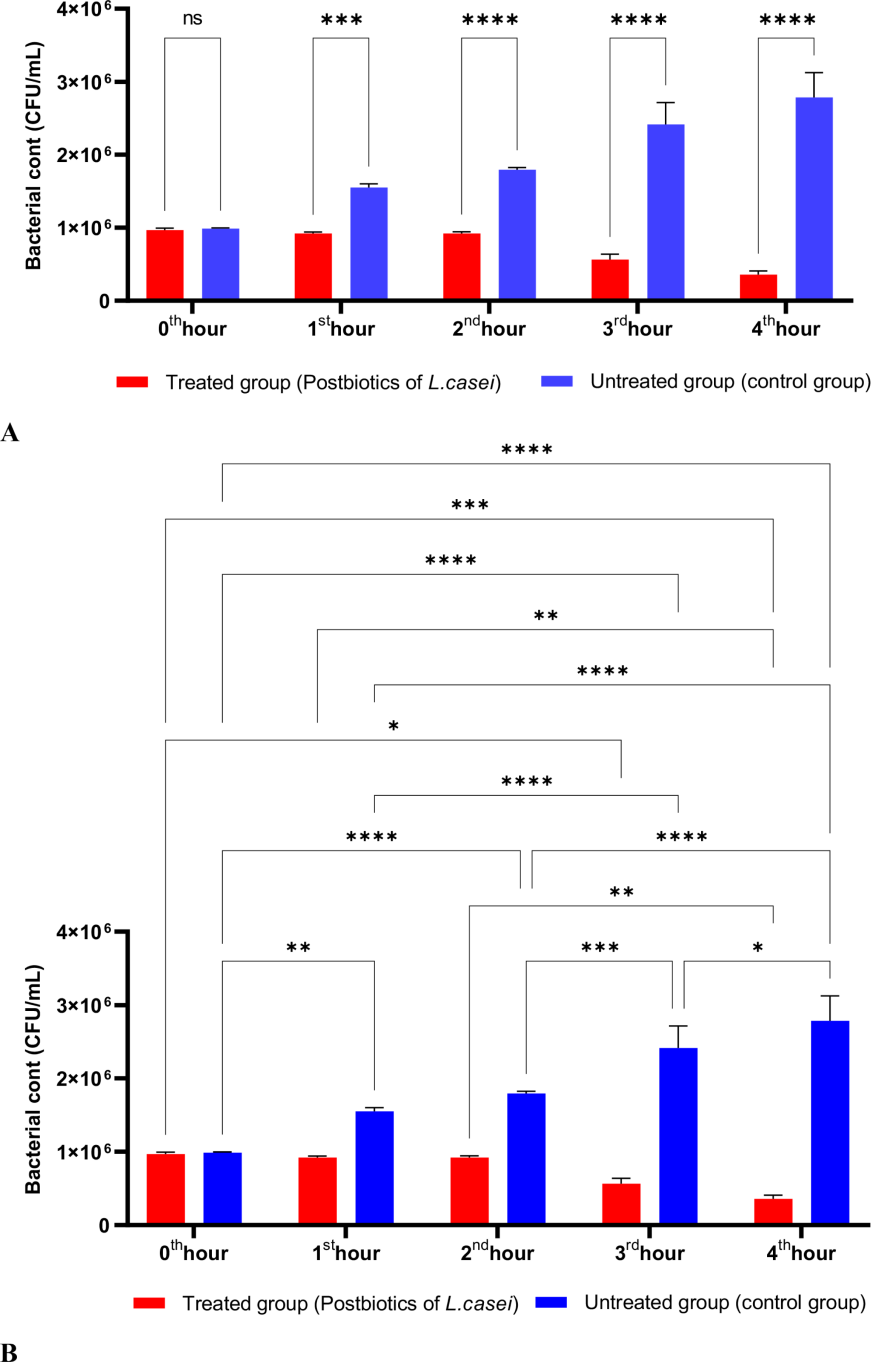
A comparative analysis of the inhibitory effects of *L. casei* postbiotics on the growth of *E. coli*, compared to the control group **(A, B)**. *p < 0.05, **p < 0.01, ***p < 0.001, ****p < 0.0001. “ns” stands for “not significant”.

When the effects of *L. casei* postbiotics on *E. coli* were studied, a significant reduction in *E. coli* was observed at the end of the first hour of incubation compared to the control group. This reduction continued to increase proportionally throughout the incubation period. A statistically significant decrease was noted when compared to the initial bacterial count (**Figures 5A, B**).

**Figure 6:**
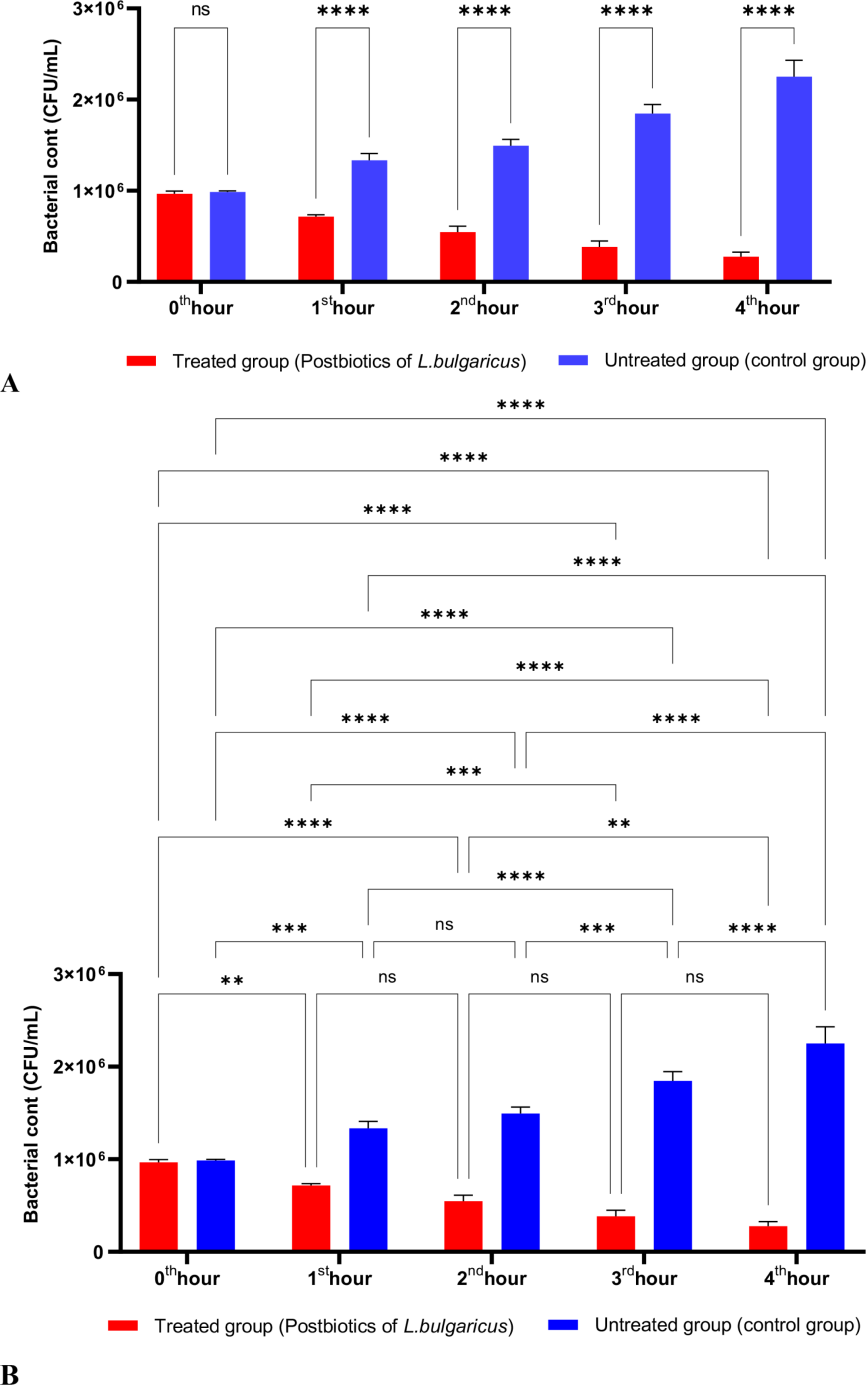
A comparative analysis of the inhibitory effects of *L. bulgaricus* postbiotics on the growth of *E. coli*, compared to the control group **(A, B)**.

A similar effect of *L. casei* on *E. coli* was observed with the postbiotics of *L. bulgaricus*. Compared to the control group, the number of *E. coli* was significantly reduced starting from the first hour of incubation. This decrease was directly proportional to the duration of incubation (**Figures 6A, B**).

**Figure 7:**
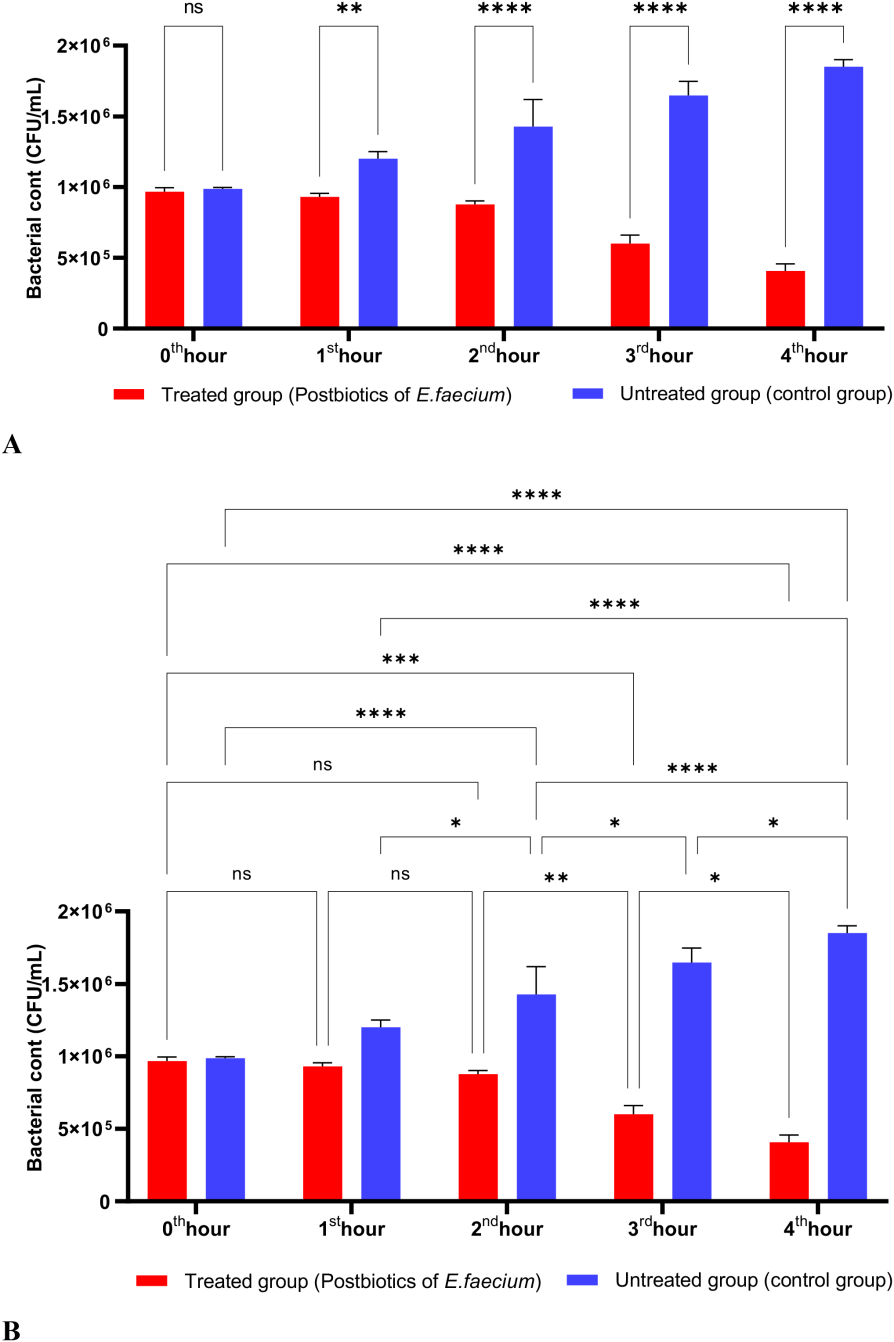
A comparative analysis of the inhibitory effects of *E. faecium* postbiotics on the growth of *E. coli*, compared to the control group **(A, B)**. *p < 0.05, **p < 0.01, ***p < 0.001, ****p < 0.0001. “ns” stands for “not significant”.

A similar effect of *L. casei* on *E. coli* was observed with the postbiotics of *L. bulgaricus*. Compared to the control group, the number of *E. coli* was significantly reduced starting from the first hour of incubation. This decrease was directly proportional to the duration of incubation (**Figures 7A, B**).

**Figure 8:**
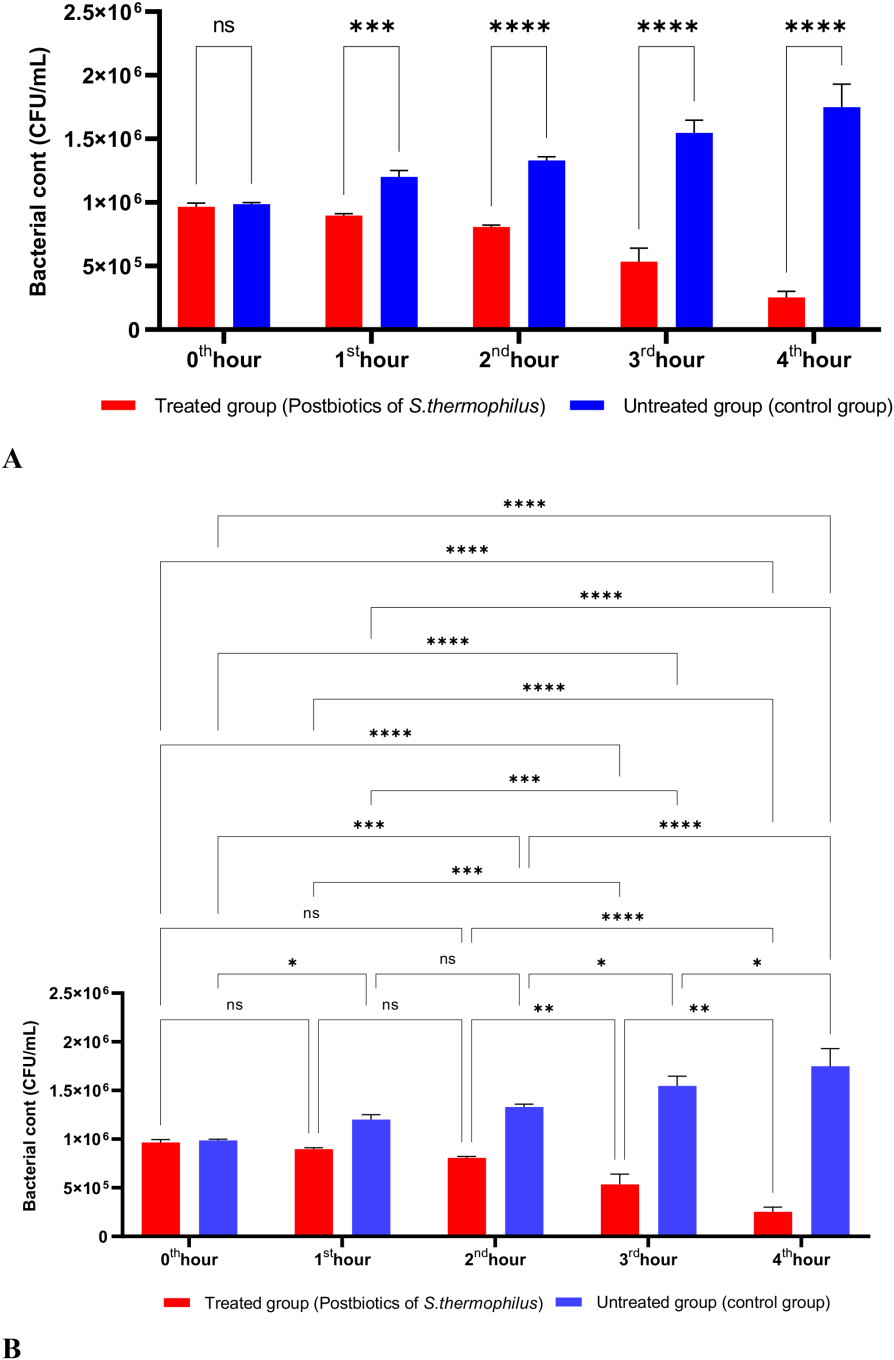
A comparative analysis of the inhibitory effects of *S. thermophilus* postbiotics on the growth of *E. coli*, compared to the control group **(A, B)**. *p < 0.05, **p < 0.01, ***p < 0.001, ****p < 0.0001. “ns” stands for “not significant”.

Research indicated that postbiotics from *S. thermophilus* were highly effective against *E. coli*. Compared to the control group, the bacterial count significantly decreased by the end of the first hour. This decrease was directly proportional to the duration of exposure (**Figures 8A, B**).

**Figure 9:**
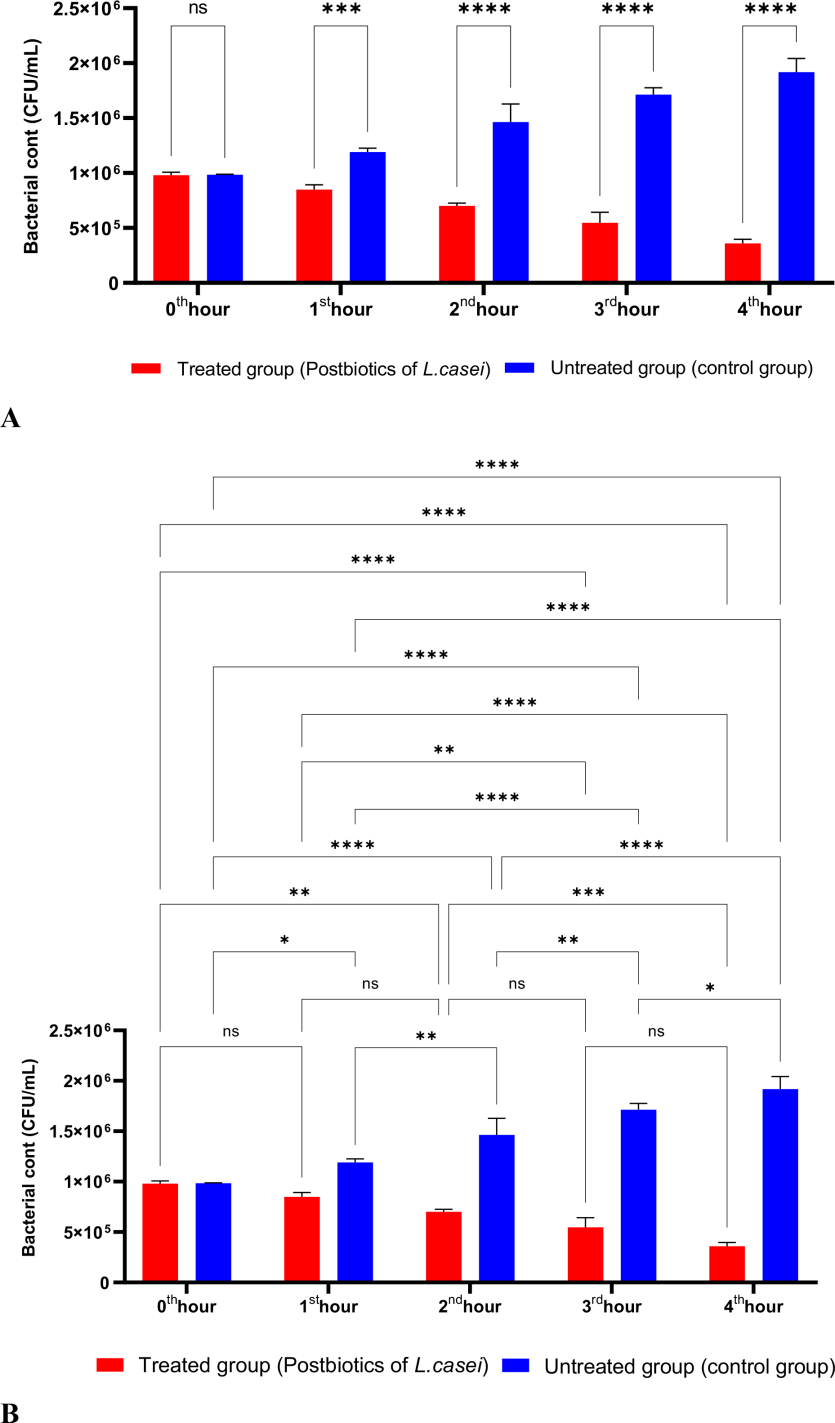
A comparative analysis of the inhibitory effects of *L. casei* postbiotics on the growth of *P. aeruginosa*, compared to the control group **(A, B)**. *p < 0.05, **p < 0.01, ***p < 0.001, ****p < 0.0001. “ns” stands for “not significant”.

*L. casei* postbiotics demonstrated significant effectiveness against *P. aeruginosa* after one hour of incubation, and this effectiveness became even more pronounced with longer incubation times (**Figures 9A, B**).

**Figure 10:**
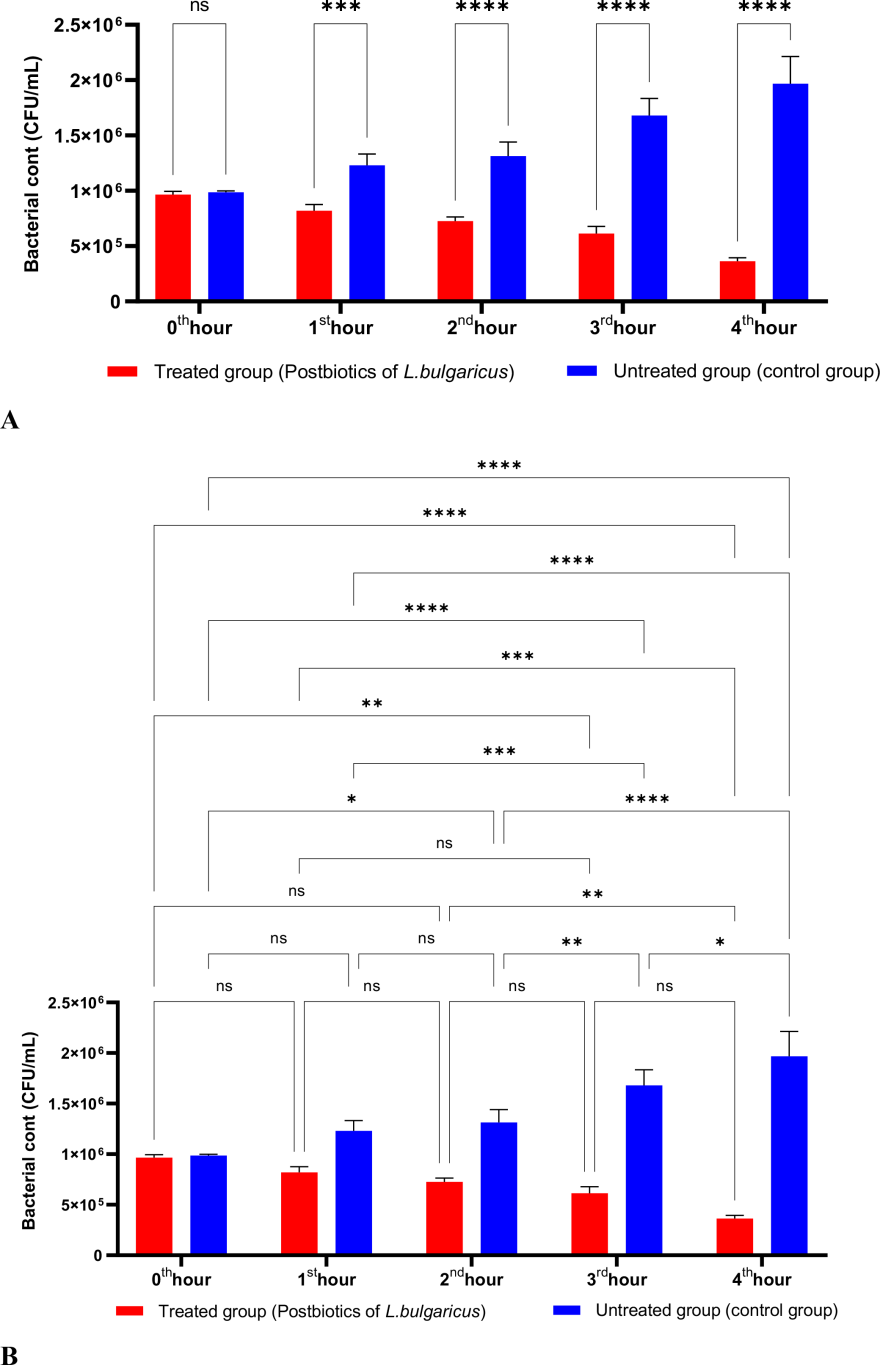
A comparative analysis of the inhibitory effects of *L. bulgaricus* postbiotics on the growth of *P. aeruginosa*, compared to the control group **(A, B)**. *p < 0.05, **p < 0.01, ***p < 0.001, ****p < 0.0001. “ns” stands for “not significant”.

Compared to the control group that lacked postbiotics, *L. bulgaricus* postbiotics were significantly effective against *P. aeruginosa* by the end of the first hour of incubation. This effectiveness increased in direct proportion to the length of the incubation period. (**Figures 10A, B**).

**Figure 11:**
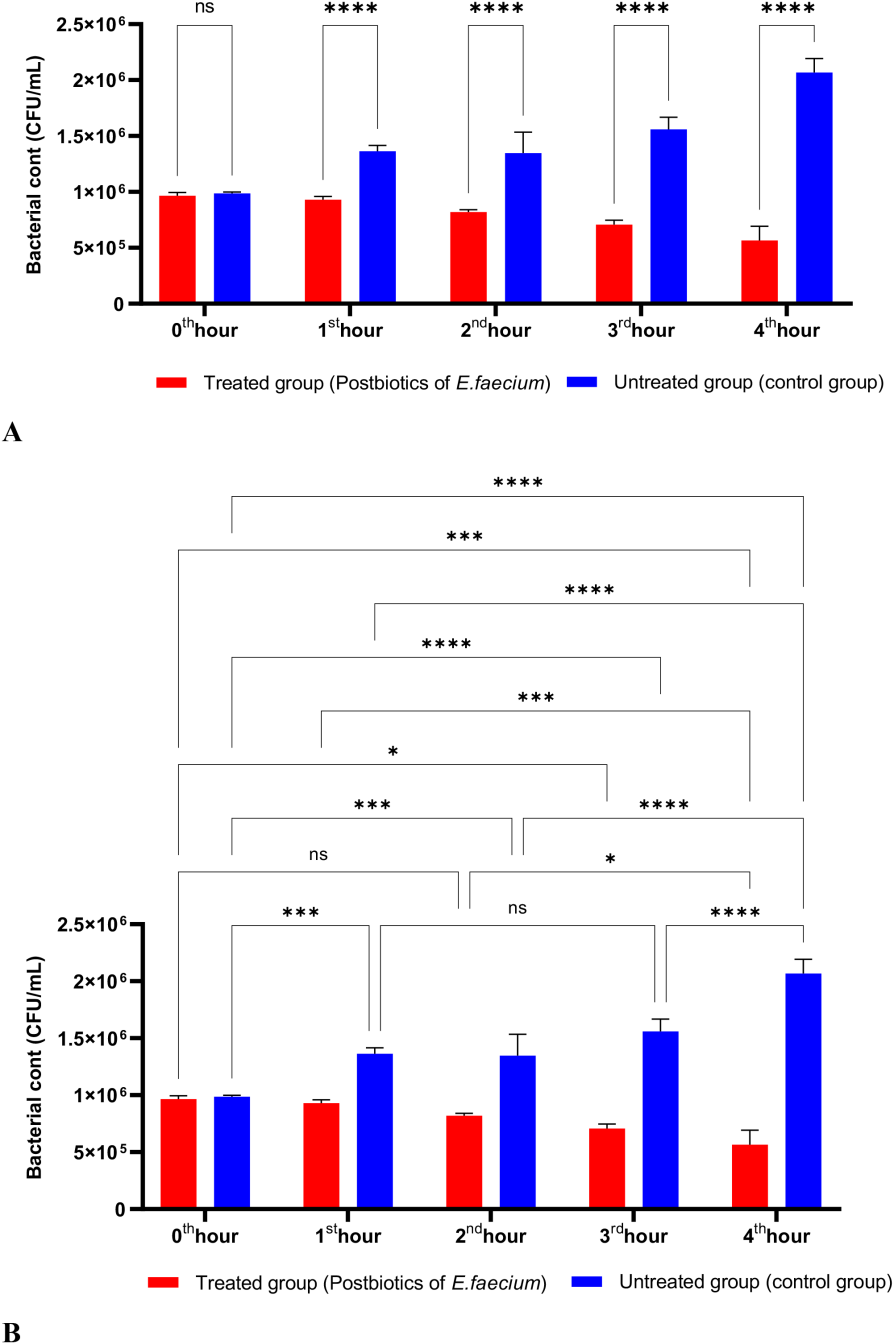
A comparative analysis of the inhibitory effects of *E. faecium* postbiotics on the growth of *P. aeruginosa*, compared to the control group **(A, B)**. *p < 0.05, ***p < 0.001, ****p < 0.0001. “ns” stands for “not significant”.

**Figure 12:**
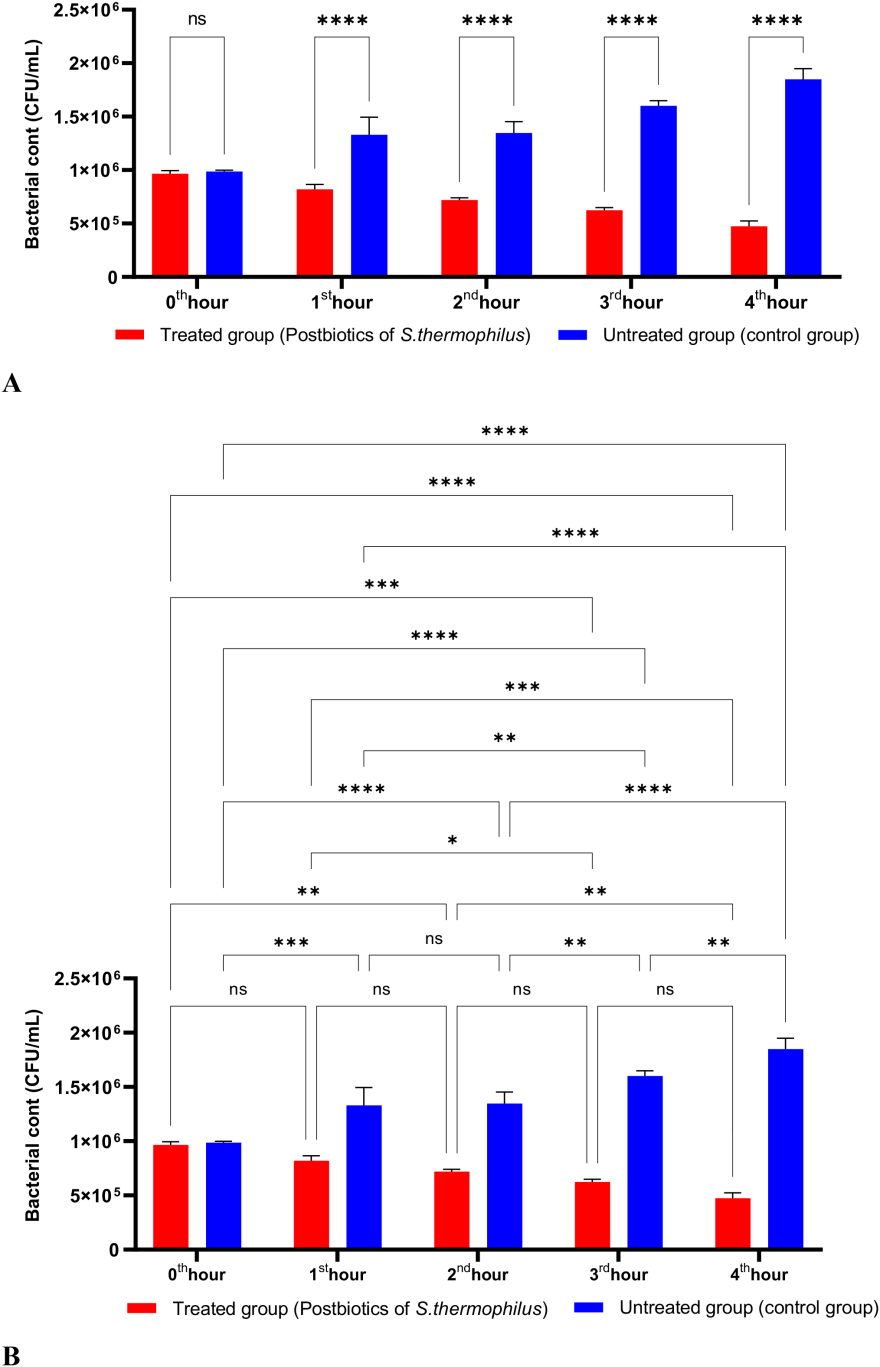
A comparative analysis of the inhibitory effects of *S. thermophilus* postbiotics on the growth of *P. aeruginosa*, compared to the control group **(A, B)**. *p < 0.05, **p < 0.01, ***p < 0.001, ****p < 0.0001. “ns” stands for “not significant”.

The activity studies found that postbiotics from *E. faecium* and *S. thermophilus* significantly inhibited the growth of *P. aeruginosa* during the first hour of incubation. Furthermore, the anti-*P. aeruginosa* activity of these postbiotics increased in direct proportion to the length of the incubation period (**Figures 11**, **12**).

**Figure 13:**
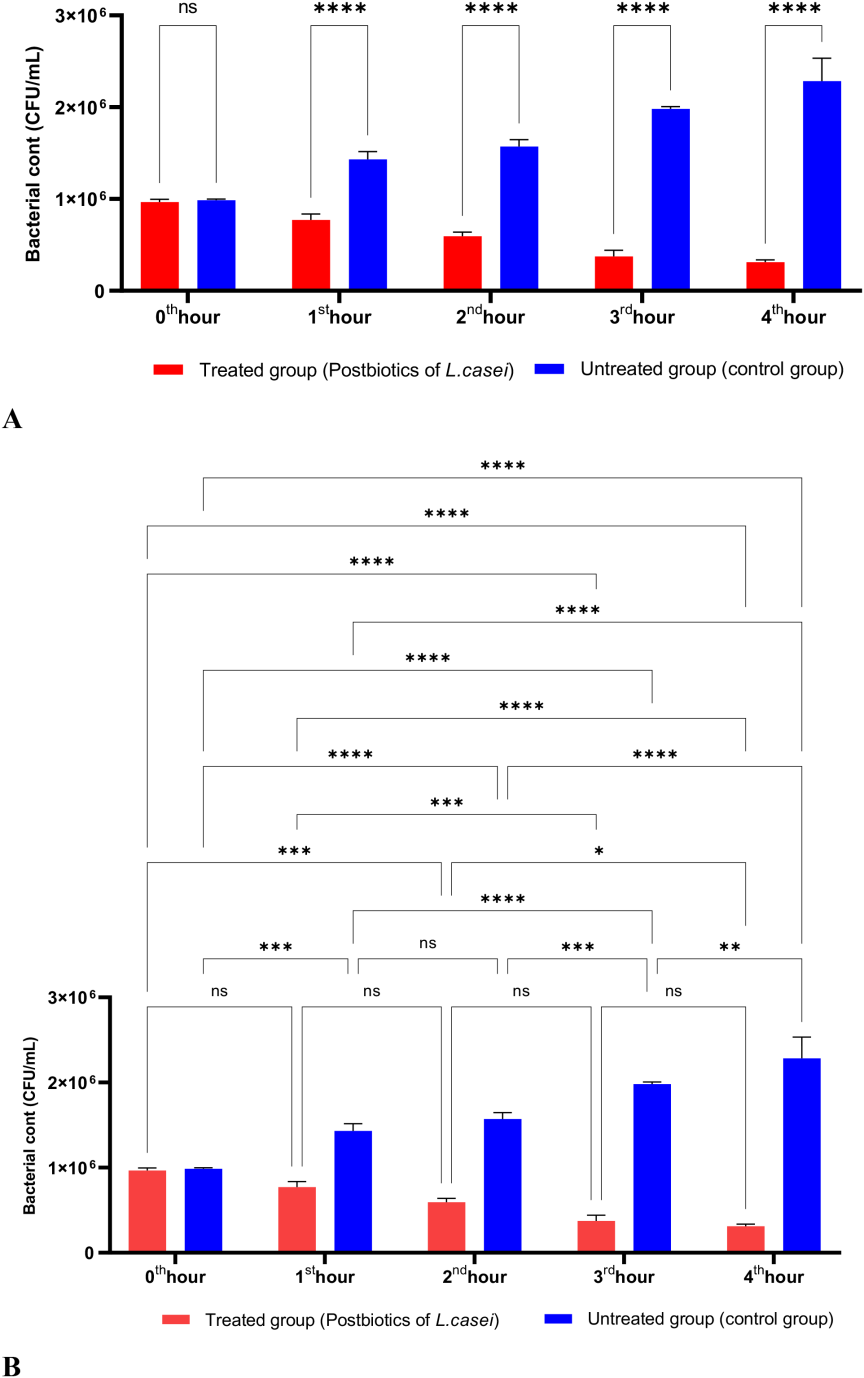
A comparative analysis of the inhibitory effects *L. casei* postbiotics on the growth of *P. aeruginosa*, compared to the control group **(A, B)**. *p < 0.05, **p < 0.01, ***p < 0.001, ****p < 0.0001. “ns” stands for “not significant”.

**Figure 14:**
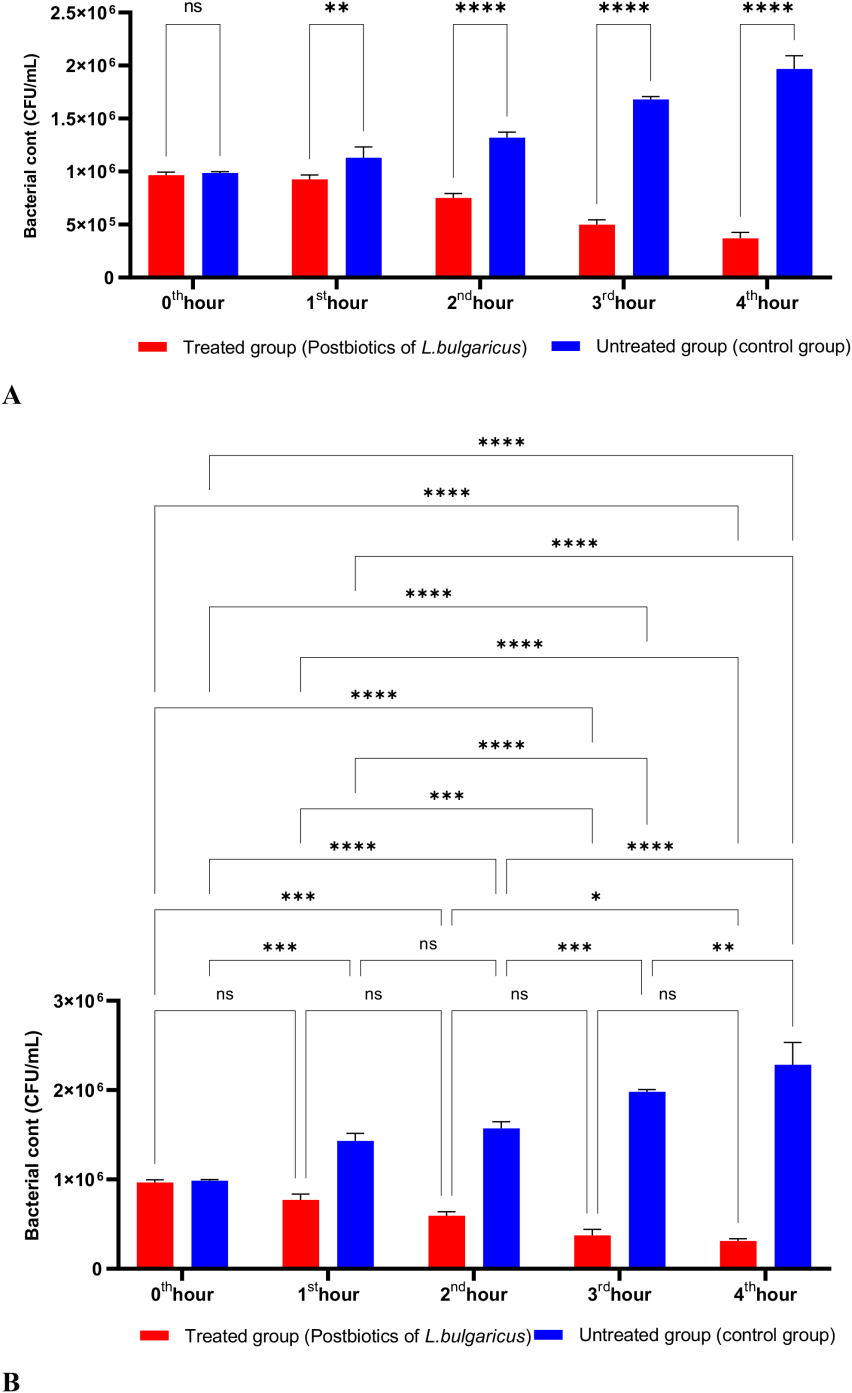
A comparative analysis of the inhibitory effects *L. bulgaricus* postbiotics on the growth of *P. aeruginosa*, compared to the control group **(A, B)**. *p < 0.05, **p < 0.01, ***p < 0.001, ****p < 0.0001. “ns” stands for “not significant”.

**Figure 15:**
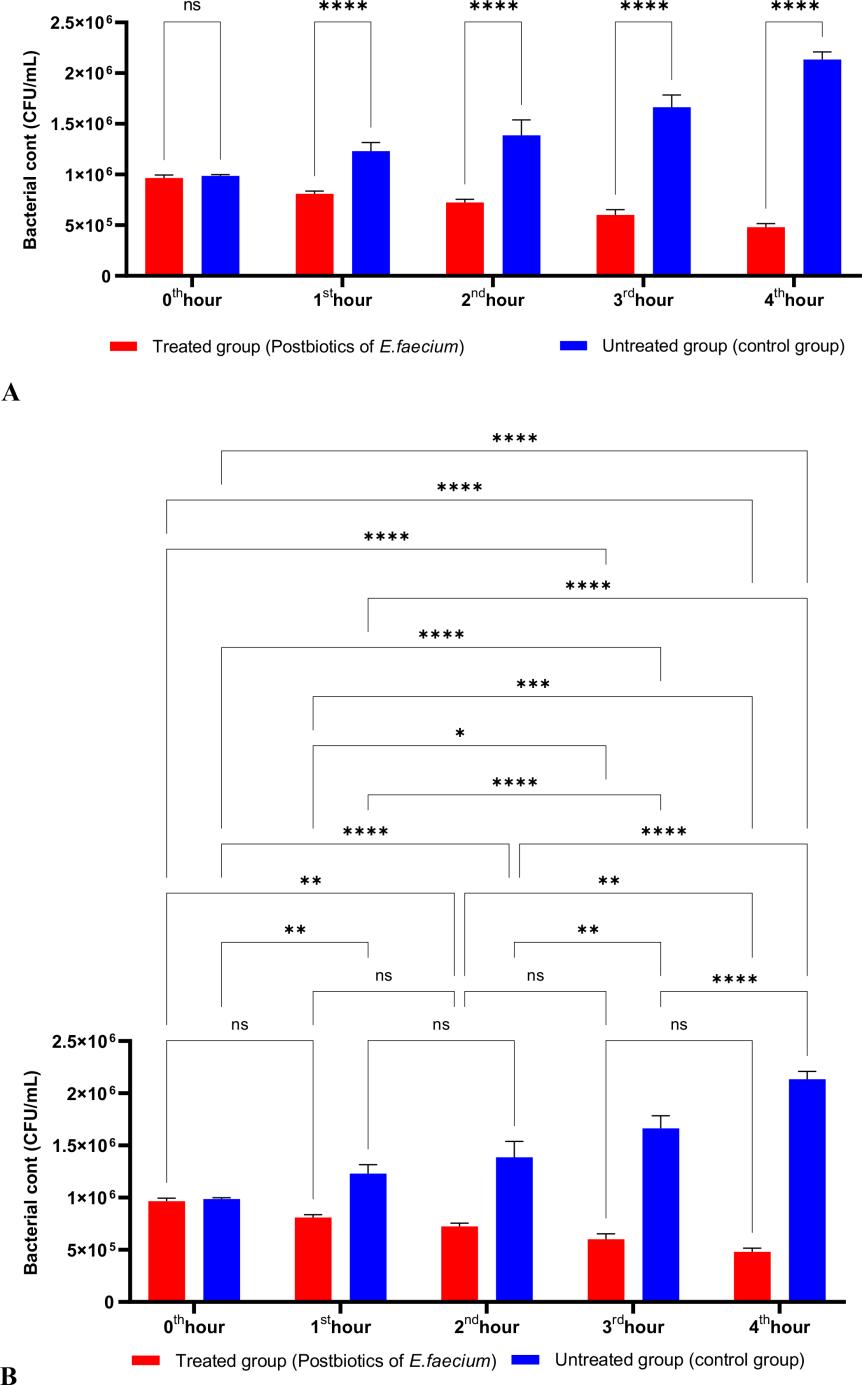
A comparative analysis of the inhibitory effects *E. faecium* postbiotics on the growth of *P. aeruginosa*, compared to the control group **(A, B)**. *p < 0.05, **p < 0.01, ***p < 0.001, ****p < 0.0001. “ns” stands for “not significant”.

**Figure 16:**
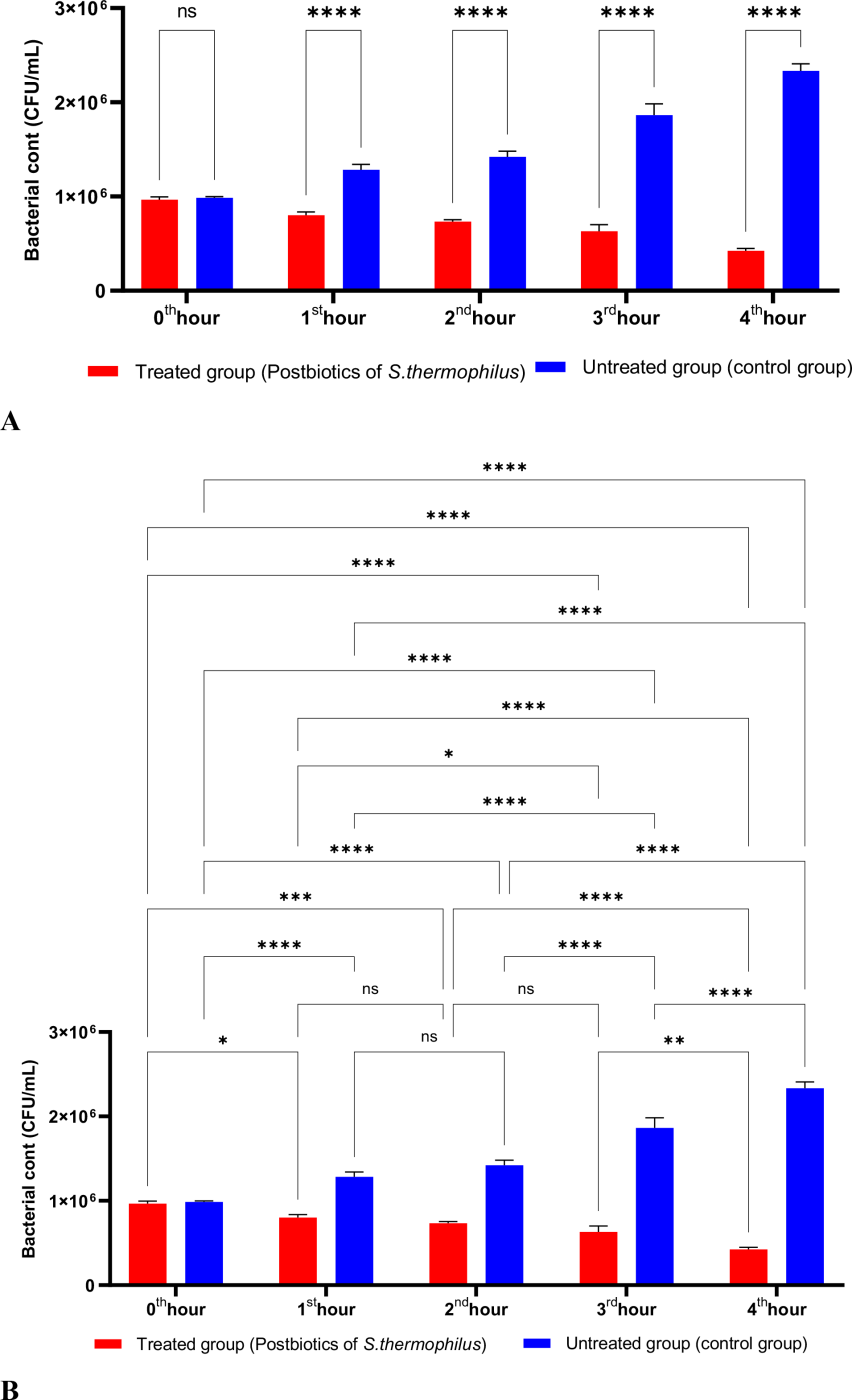
A comparative analysis of the inhibitory effects *E. faecium* postbiotics on the growth of *P. aeruginosa*, compared to the control group **(A, B)**. *p < 0.05, **p < 0.01, ***p < 0.001, ****p < 0.0001. “ns” stands for “not significant”.

The activities of *L. casei*, *L. bulgaricus*, *E. faecium*, and S*. thermophilus* postbiotics against *Proteus mirabilis*, along with the control group (medium without any postbiotics), are illustrated in **Figures 12**–**16**. After one hour of incubation, it was found that the postbiotics from *L. bulgaricus* significantly inhibited the growth of *P. mirabilis*. However, the anti-*P.mirabilis* activities of the postbiotics from *L. casei*, *E. faecium*, and *S. thermophilus* were statistically more effective than those of *L. bulgaricus* (**Figures 13**-**16**).

**Figure 17:**
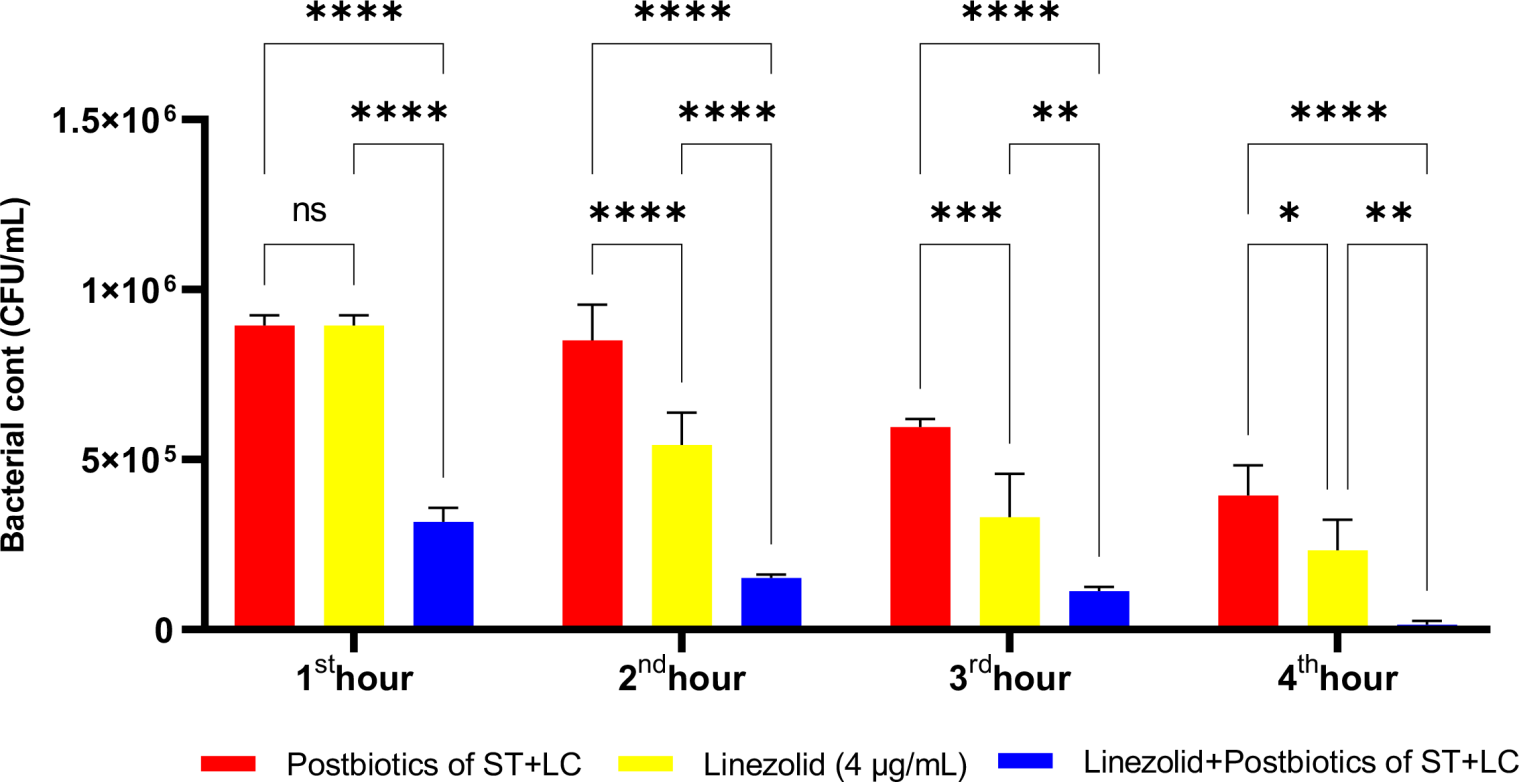
Comparative evaluation of the efficacy against *S. aureus* of the combination of *S. thermophilus* and *L. casei* postbiotics, linezolid treatment alone, and the combination of amikacin with postbiotics. *p < 0.05, **p < 0.01, ***p < 0.001, ****p < 0.0001. “ns” stands for “not significant”.

All experiments standardized the initial bacterial inoculum to ensure equal starting conditions. **Figure 17** presents the antibacterial effects of amikacin, the *S. thermophilus* and *L. casei* postbiotic combination (ST+LC), and the linezolid-postbiotic combination against *S. aureus*. The ST+LC postbiotic combination did not exhibit a statistically significant effect on bacterial growth at the first hour of incubation (p<0.99). However, the linezolid-postbiotic combination (ST+LC + linezolid) demonstrated a statistically significant inhibition of bacterial growth starting from the first hour of incubation. The ST+LC postbiotic combination significantly reduced bacterial growth from the second and third hours of incubation. The most remarkable finding was that using the ST+LC postbiotic combination with linezolid significantly inhibited bacterial growth from the first hour of incubation (**Figure 17**; p<0.0001).

**Figure 18:**
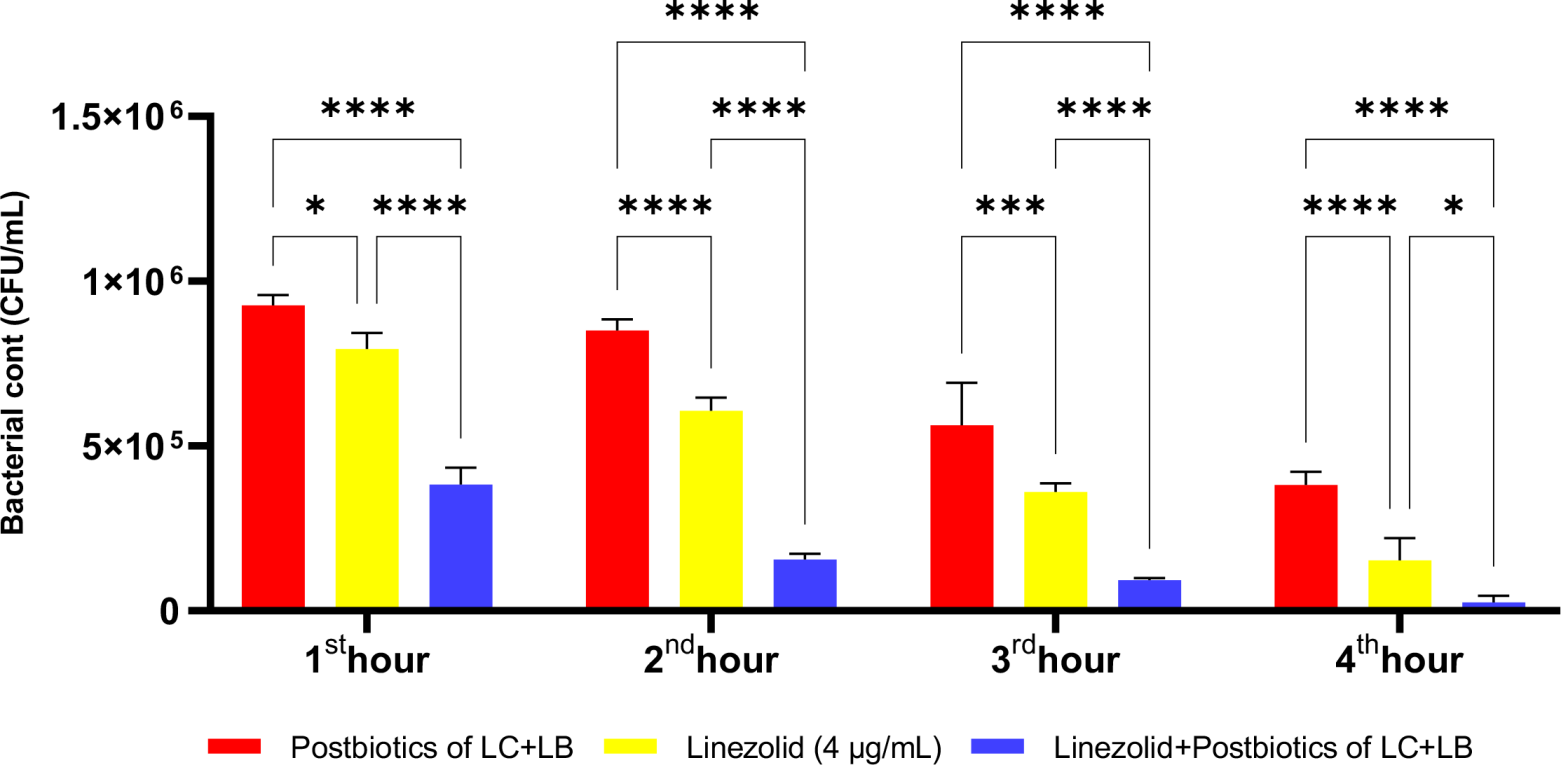
Comparative evaluation of the efficacy against *S. aureus* of the combination of *L. casei* and *L. bulgaricus* postbiotics, linezolid treatment alone, and the combination of amikacin with postbiotics. *p < 0.05, ***p < 0.001, ****p < 0.0001. “ns” stands for “not significant”.

In the experiments, at the end of the first hour of incubation, the antibacterial efficacy of linezolid was found to be statistically significantly higher compared to the *L. casei* and *L. bulgaricus* postbiotic combination (LC+LB). However, the combination therapy was significantly more effective than the linezolid-plus-postbiotic combination (linezolid+LC+LB) when comparing the efficacy of amikacin against *Staphylococcus aureus*. At the end of all incubation periods, the triple combination (linezolid + LC + LB postbiotic combination) was statistically significantly more effective than linezolid alone (**Figure 18**; p<0.018).

**Figure 19:**
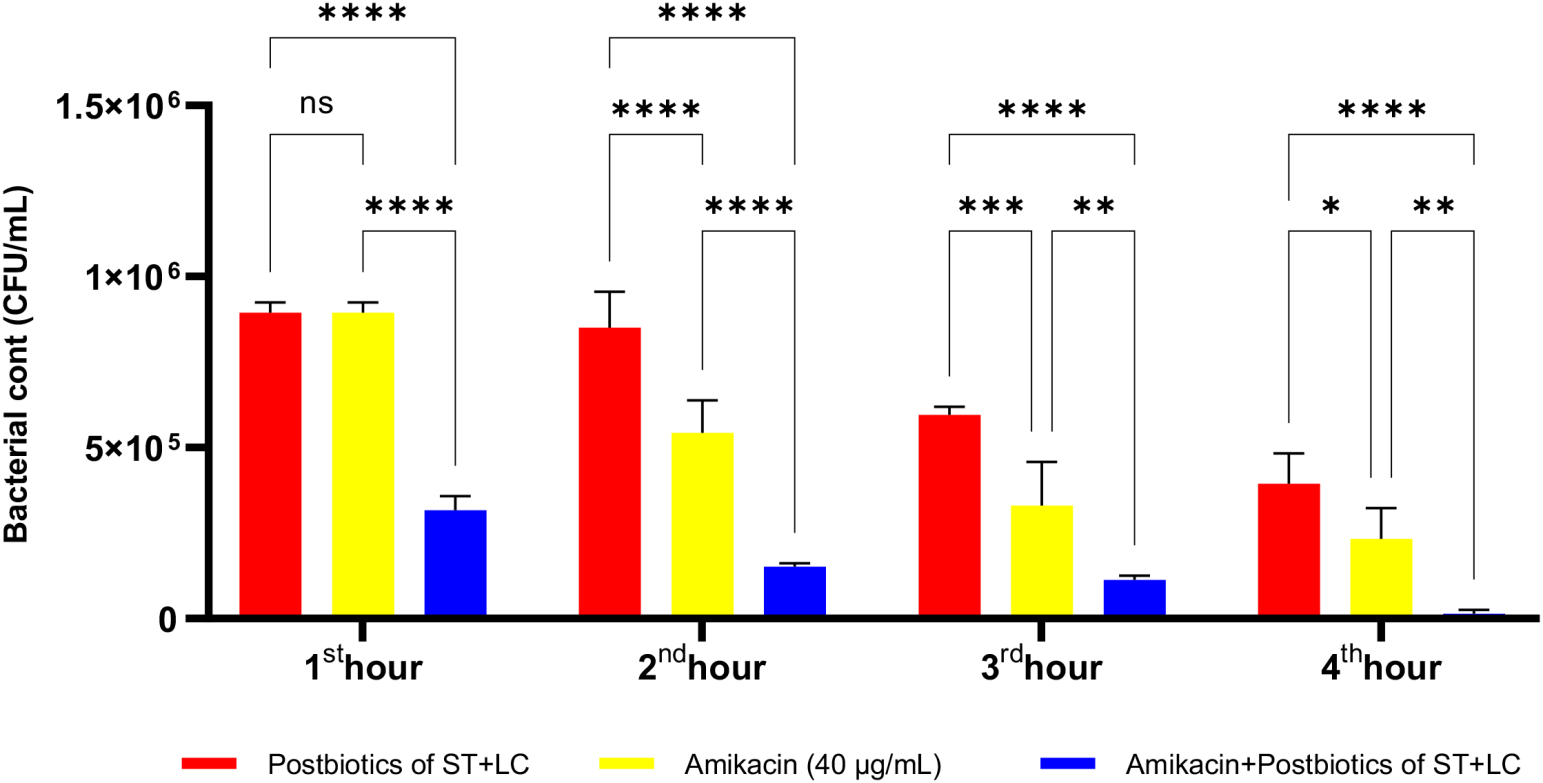
Comparative evaluation of the efficacy against *E. coli* of the combination of *S. thermophilus* and *L. bulgaricus* postbiotics, amikacin treatment alone, and the combination of amikacin with postbiotics. *p < 0.05, **p < 0.01, ***p < 0.001, ****p < 0.0001. “ns” stands for “not significant”.

At the end of the first hour of incubation, there was no statistically significant difference in the efficacy of the postbiotic combination (ST+LC) compared to amikacin against *E. coli*, as shown in **Figure 19** (p<0.509). However, similar to the results observed with *Staphylococcus aureus*, the antibacterial efficacy of the amikacin-postbiotic combination against *E. coli* was significantly more potent than that of the other two groups. Additionally, this antibacterial effect was found to increase proportionally with the length of the incubation period (**Figure 19**).

**Figure 20:**
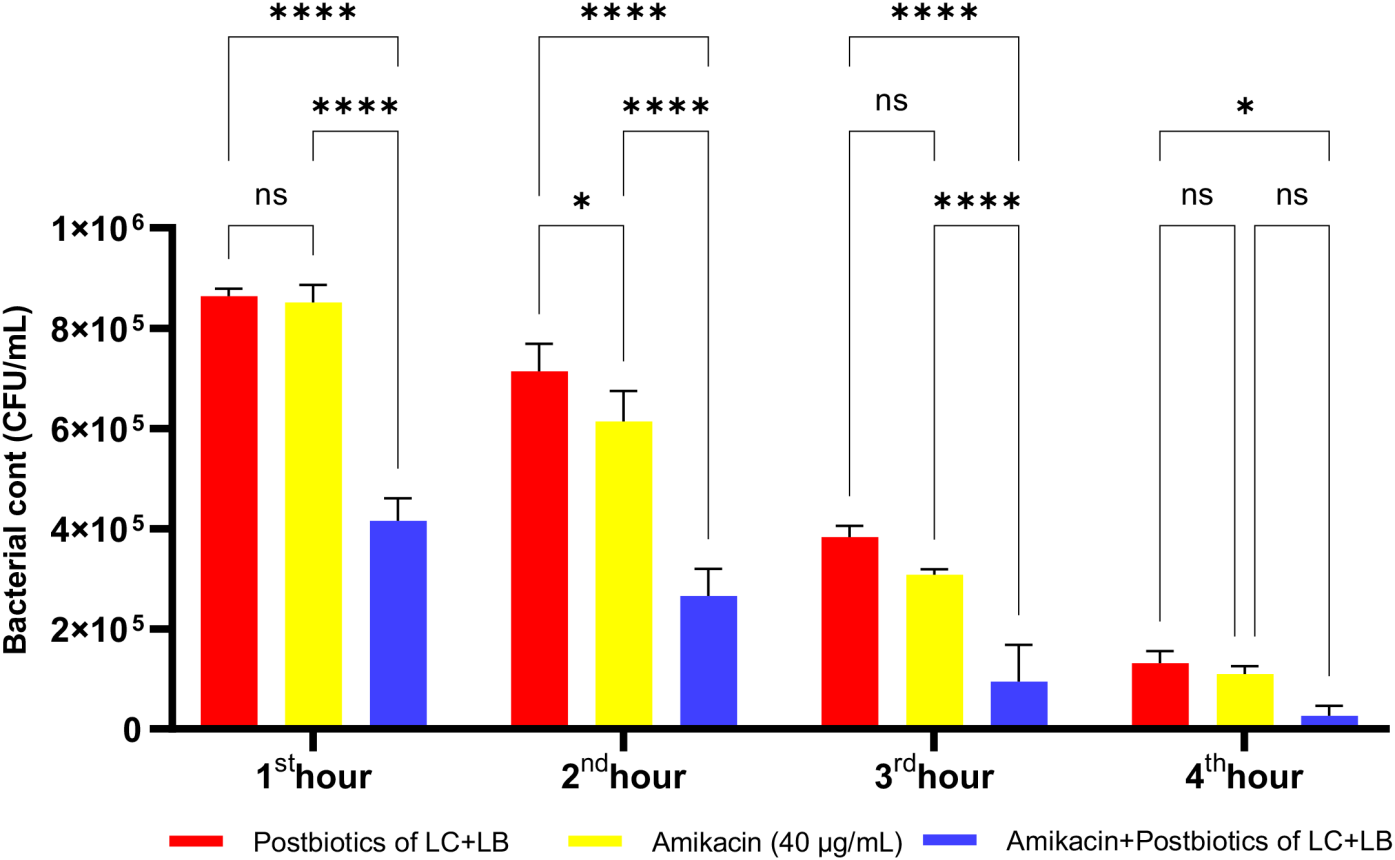
Comparative evaluation of the efficacy against *E. coli* of the combination of *L. casei* and *L. bulgaricus* postbiotics, amikacin treatment alone, and the combination of amikacin with postbiotics. *p < 0.05, ****p < 0.0001. “ns” stands for “not significant”.

As shown in **Figure 20**, at the end of the second hour of incubation, amikacin’s antibacterial efficacy against *E. coli* was higher than that of the postbiotic combination (ST+LC; p<0.017). However, while this effect was statistically significant at the second hour of incubation, the difference between the groups was no longer statistically significant at later incubation periods. Moreover, from the end of the first hour onward, the amikacin-postbiotic combination (LC+LB) treatment group demonstrated a statistically significantly more substantial antibacterial effect than either the postbiotic (LC+LB) treatment group alone or the amikacin treatment group alone (**Figure 20**).

**Figure 21:**
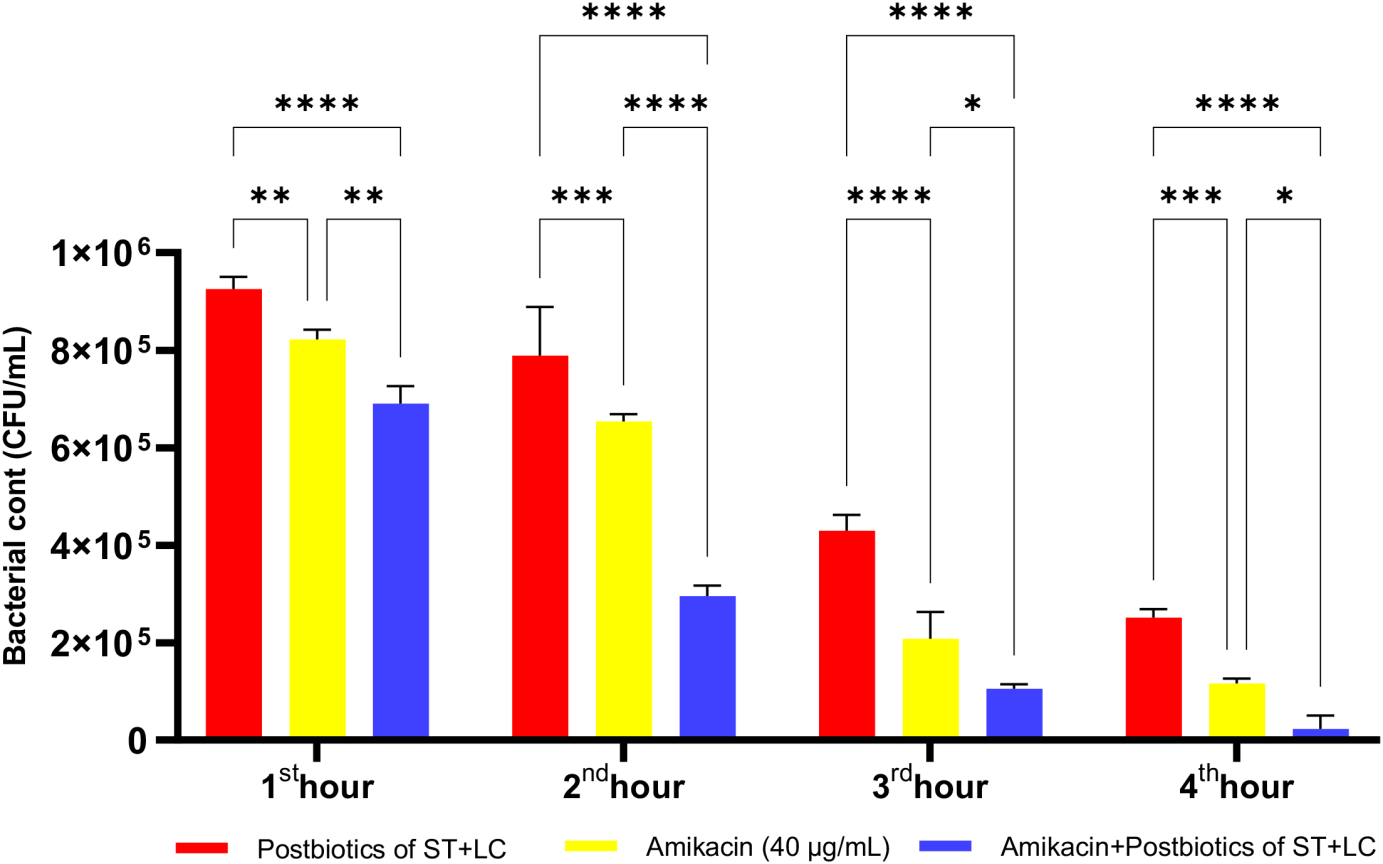
Comparative evaluation of the efficacy against *P. aeruginosa* of *S. thermophilus* and *L. casei* postbiotics, amikacin treatment alone, and the combination of amikacin with postbiotics. *p < 0.05, **p < 0.01, ***p < 0.001, ****p < 0.0001. “ns” stands for “not significant”.

Amikacin significantly inhibited the growth of *P. aeruginosa* from the first hour of incubation, demonstrating a statistically significant antibacterial effect. At the end of the first hour of incubation, the amikacin treatment group exhibited a more potent antibacterial effect than the postbiotic combination (ST+LC) treatment group (p<0.009). Additionally, the triple combination treatment group (Amikacin + ST + LC) demonstrated greater efficacy against *P. aeruginosa* compared to the amikacin treatment group alone. At the end of the second hour, the triple combination treatment group exhibited a more potent antibacterial effect than amikacin treatment alone. However, in the later incubation periods, the statistical significance of the difference in efficacy between the triple combination treatment group and the amikacin treatment group decreased (**Figure 21**; p<0.020).

**Figure 22:**
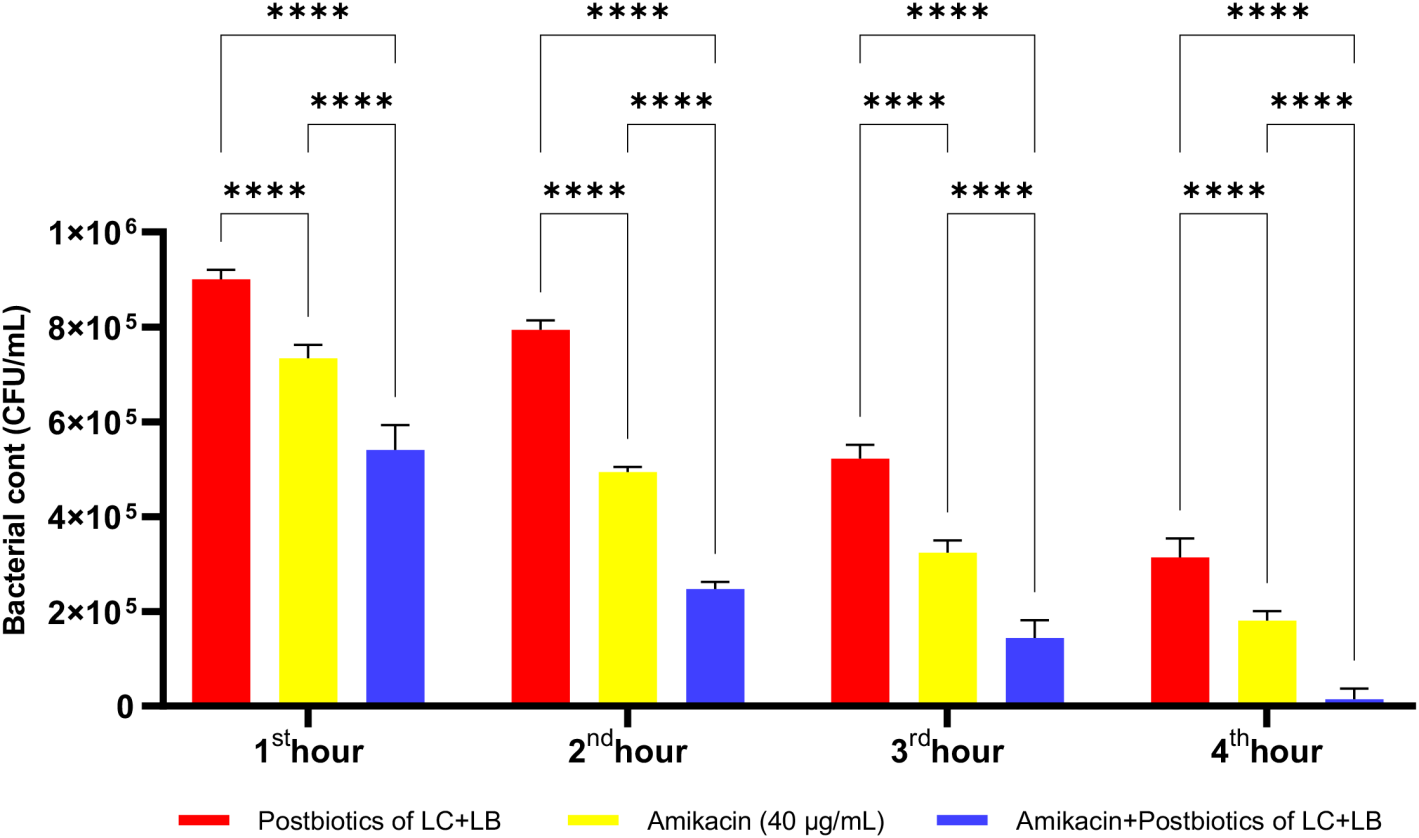
Comparative evaluation of the efficacy against *P. aeruginosa* of *L. casei* and *L. bulgaricus* postbiotics, amikacin treatment alone, and the combination of amikacin with postbiotics. ****p < 0.0001.

As shown in **Figure 22**, the antibacterial efficacy of amikacin against *P. aeruginosa* was statistically significantly higher compared to the postbiotic (LC+LB) treatment group (p<0.0001). More importantly, the antibacterial efficacy of the triple treatment group (amikacin + *L. casei* + *L. bulgaricus*) against *P. aeruginosa* was significantly more potent than that of amikacin treatment alone. This effect remained consistently high throughout all incubation periods, starting from the end of the first hour of incubation (**Figure 22**; p<0.0001).

**Figure 23:**
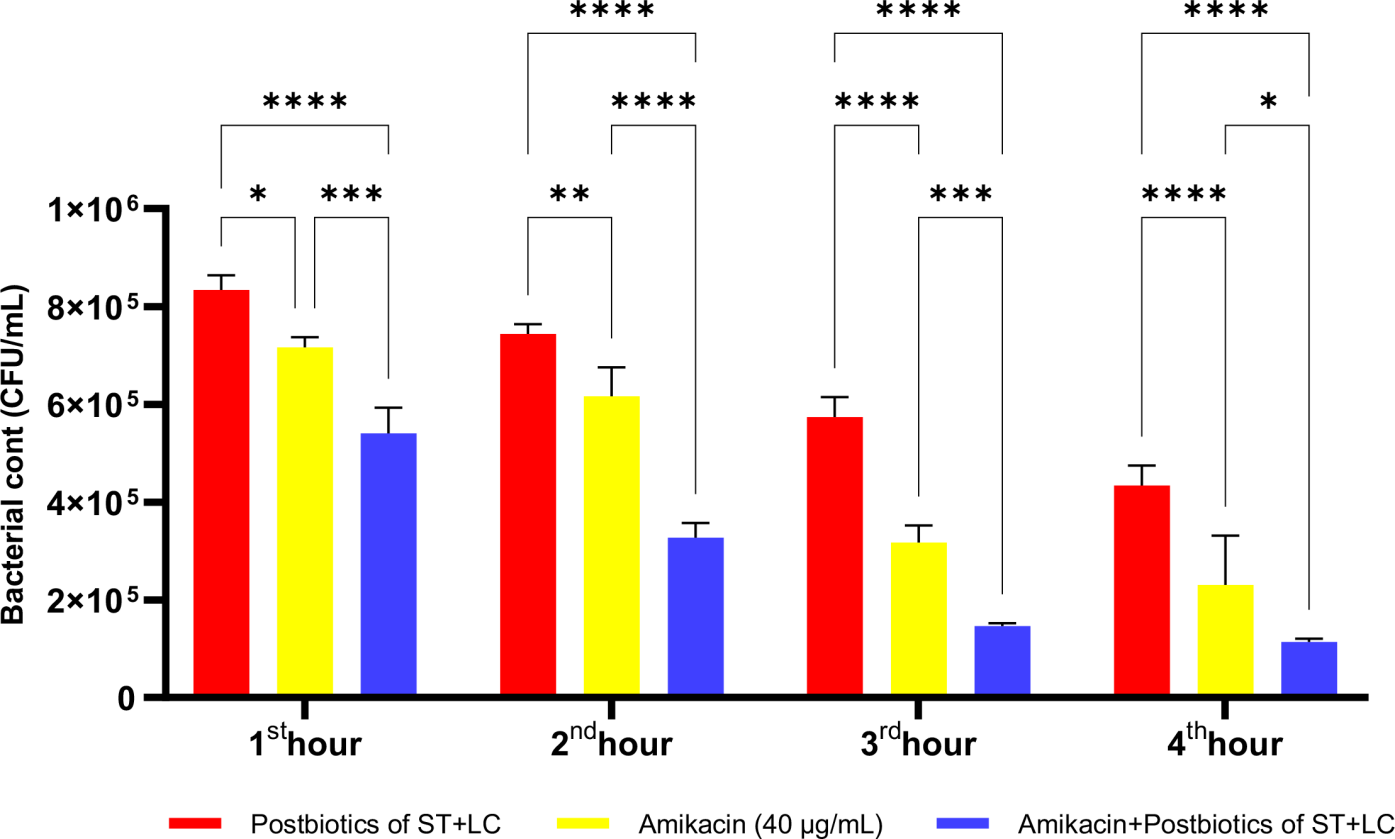
Comparative evaluation of the efficacy against *P. mirabilis* of *S. thermophilus* and *L. casei* postbiotics, amikacin treatment alone, and the combination of amikacin with postbiotics. *p < 0.05, **p < 0.01, ***p < 0.001, ****p < 0.0001. “ns” stands for “not significant”.

The postbiotic combination of *S. thermophilus* and *L. casei* exhibited a marked antibacterial effect against *P. mirabilis*. Compared to the amikacin-treated group, the postbiotic combination demonstrated significantly greater antibacterial activity, as indicated by viable cell counts at each hourly time point. Moreover, the triple therapy group, in which postbiotics were co-administered with amikacin, showed significantly enhanced efficacy compared to amikacin alone (**Figure 23**; p<0.01).

**Figure 24:**
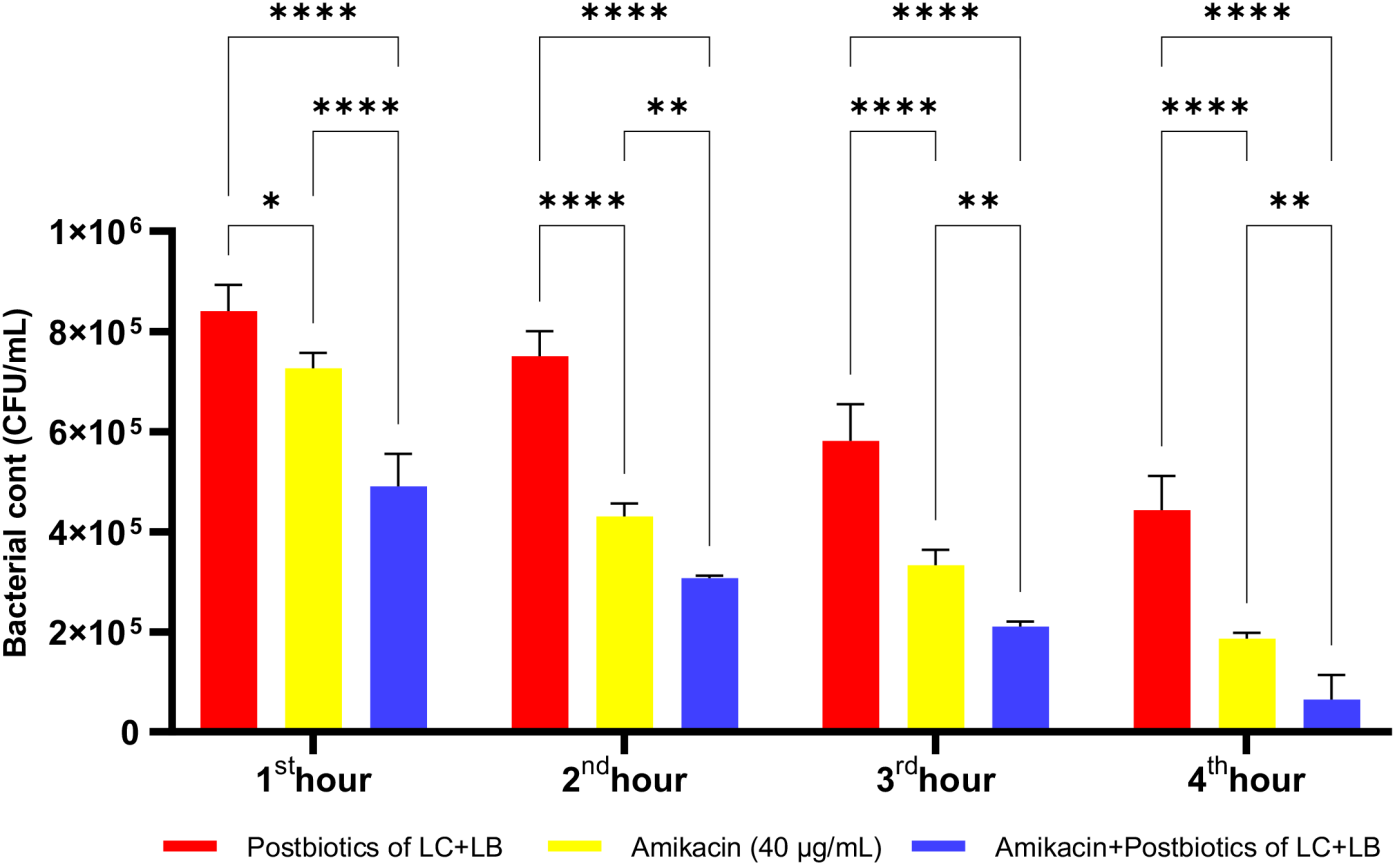
Comparative evaluation of the efficacy against *P. mirabilis* of *L. casei* and *L. bulgaricus* postbiotics, amikacin treatment alone, and the combination of amikacin with postbiotics. *p < 0.05, **p < 0.01, ****p < 0.0001.

The LC+LB postbiotic combination exhibited vigorous time-dependent antibacterial activity against *P. mirabilis*. Although the standard antibiotic amikacin demonstrated significantly higher efficacy compared to the postbiotic combination, the latter still achieved a notable level of antibacterial effect. However, when the postbiotic combination was administered together with amikacin as a triple therapy, the reduction in bacterial load was markedly diminished, suggesting a potential antagonistic interaction (**Figure 24**; p<0.008; **Table 1**).

**Table 1:** Summary of synergistic effects observed between postbiotics and antibiotics across different bacterial strains and timepoints.

Bacterial strain	Postbiotic combination	Antibiotic	Time (h)	Synergistic effect	P Value
*S. aureus*	*S. thermophilus + L. casei*	Linezolid	1	No	>0.9999
			2	Strong synergy	<0.0001
			3	Moderate synergy	0.0002
			4	Weak synergy	0.0216
*E. coli*	*S. thermophilus + L. casei*	Amikacin	1	Strong synergy	<0.0001
			2	Strong synergy	<0.0001
			3	Moderate synergy	0.0003
			4	Moderate synergy	0.0070
*E. coli*	*L. casei + L. bulgaricus*	Amikacin	1	No	0.9290
			2	Weak synergy	0.0177
			3	No	0.0870
			4	Weak synergy	0.0126
*P. aeruginosa*	*S. thermophilus + L. casei*	Amikacin	1–4	Strong synergy	<0.0001
*P. aeruginosa*	*L. casei + L. bulgaricus*	Amikacin	1–4	Strong synergy	<0.0001
*P. mirabilis*	*S. thermophilus + L. casei*	Amikacin	1	Weak synergy	0.0106
			2	Strong synergy	<0.0001
			3	Strong synergy	<0.0001
			4	Moderate synergy	0.0110
*P. mirabilis*	*L. casei + L. bulgaricus*	Amikacin	1	Weak synergy	0.0146
			2	Strong synergy	<0.0001
			3	Strong synergy	0.0078
			4	Strong synergy	0.0086

The statistical significance (P values) is indicated based on Tukey’s multiple comparisons test.

## Discussion

Antimicrobial resistance (AMR) poses a growing threat to global health systems. In this context, postbiotics have attracted attention as alternative or complementary treatment strategies, especially against multidrug-resistant pathogens. In this study, the antimicrobial activities of postbiotics produced by probiotic microorganisms, including *L. casei*, *L. bulgaricus*, *E. faecium*, and *S. thermophilus*, were evaluated against essential pathogens such as *Staphylococcus aureus, E. coli, P. aeruginosa*, and *P. mirabilis* (Ribeiro et al., 2024; Pérez-López et al., 2025).

The increasing prevalence of AMR worldwide has heightened the need for innovative treatment strategies beyond traditional antibiotics. This study has demonstrated that postbiotics from *L. casei, L. bulgaricus, E. faecium*, and *S. thermophilus* exhibit significant antimicrobial effects against nosocomial pathogens, including *S. aureus, E. coli, P. aeruginosa*, and *P. mirabilis*. More importantly, combining these postbiotics with amikacin, one of the aminoglycoside antibiotics, showed a statistically significant synergistic effect compared to using either agent alone (Ribeiro et al., 2024).

The findings demonstrate that postbiotics can significantly inhibit the growth of pathogens when used alone or in combination with antibiotics. In particular, combining *L. casei* and *S. thermophilus* postbiotics with antibiotics (linezolid and amikacin) showed a synergistic effect against *S. aureus* and *E. coli*. This suggests that postbiotics may enhance the efficacy of antibiotics, allowing for the use of lower doses.

Studies in the literature on postbiotics’ antimicrobial, anti-inflammatory, and immunomodulatory properties reveal that these components are promising agents in infection control. Additionally, the stability of postbiotics and the absence of live microorganisms support their safe use, particularly in immunocompromised individuals (Ji et al., 2023; Mazziotta et al., 2023).

Postbiotics contain bioactive metabolites such as short-chain fatty acids, antimicrobial peptides, and enzymes and are notable for their capacity to modulate host immunity and exhibit direct antimicrobial activity. The findings obtained in this study support this two-way effect at an experimental level. *In vitro* experiments showed that postbiotics suppress bacterial proliferation from the early hours and do this without causing cell toxicity. This indicates that postbiotics are safe and biocompatible treatment agents (Ünal et al., 2024; Ranjbar et al., 2025; Tenea et al., 2025).

Among the postbiotics tested in experiments, *S. thermophilus* and *E. faecium* showed remarkable antimicrobial effects, primarily on *S. aureus* and *P. aeruginosa*. Over time, the increasing impacts of these postbiotics provide clues that they interfere with bacterial replication kinetics or quorum sensing mechanisms (Doe et al., 2025; Sudheer et al., 2025).

The study’s most striking findings were obtained with combination treatments. Postbiotics and antibiotics significantly enhanced antimicrobial activity against resistant pathogens, including *S. aureus, E. coli*, and *P. aeruginosa*. In some combinations (for example, *L. casei* + *L. bulgaricus* + amikacin), bacterial inhibition was observed from the first hour of incubation. It was statistically significant compared to the treatment groups alone.

This synergistic effect is explained by the fact that postbiotics increase cell wall permeability, prevent biofilm formation, or weaken bacterial defense responses. Thus, the antibiotic’s entry into the cell becomes easier, and its effect increases (Garg et al., 2024; Zhang et al., 2025).

Additionally, the data obtained revealed that different bacterial strains exhibit distinct sensitivities to postbiotics. For example, combinations of *L. casei* and *E. faecium* showed higher efficacy on *P. mirabilis*. This suggests that personalized postbiotic-antibiotic treatments may be possible, depending on the pathogen profile.

Although the results obtained are promising, this study was conducted *in vitro*. Therefore, it is essential to evaluate the efficacy and safety of postbiotics in *in vivo* models, incorporating pharmacokinetic analyses, and utilizing a diverse range of clinical strains to understand their translational value. Additionally, the molecular mechanisms underlying the synergistic effects of postbiotics require elucidation (Garg et al., 2024; Zhang et al., 2024; Smith et al., 2025; Zhang et al., 2025).

## Conclusion

This study demonstrated that postbiotics derived from probiotic microorganisms, including *Lacticaseibacillus casei*, *Lactobacillus bulgaricus*, *Enterococcus faecium*, and *Streptococcus thermophilus*, exhibit significant antimicrobial activity against resistant nosocomial pathogens. Notably, time-dependent and statistically significant inhibition was observed when postbiotics were applied alone and in combination with antibiotics, particularly against *Staphylococcus aureus, Escherichia coli, Pseudomonas aeruginosa*, and *Proteus mirabili*s.

The synergy observed between postbiotics and amikacin resulted in enhanced antibacterial efficacy compared to the antibiotic alone, highlighting the potential of postbiotics as adjuvant agents in antimicrobial therapy. This synergistic interaction may represent a novel model in infection management, particularly in light of the current stagnation in antibiotic development.

Furthermore, the low cytotoxicity profile of postbiotics, as validated through MTT assays, and their ability to inhibit pathogens without harming host cells underscore their safety and biological compatibility. The observed strain-specific susceptibility patterns may also support the development of microbiome-guided, precision-based antimicrobial approaches.

In summary, the findings provide robust evidence that postbiotics, whether used alone or in combination with conventional antibiotics, may serve as effective, innovative, and sustainable therapeutic alternatives in the fight against antimicrobial resistance. Future *in vivo* and clinical studies will be critical to confirm their translational applicability and pave the way for their integration into routine clinical practice.

## Data Availability

The original contributions presented in the study are included in the article/**Supplementary Material**. Further inquiries can be directed to the corresponding author.

## Supplementary material

Supplementary Figure 1 Workflow of the methodology applied in this study, including postbiotic extraction, quantification (Bradford Assay), antimicrobial combination testing (binary and ternary mixtures), and cytotoxicity assay (MTT-based evaluation on Vero cells).
