# Correction: Synergistic antibacterial effects of postbiotics combined with linezolid and amikacin against nosocomial pathogens

**DOI:** 10.3389/fcimb.2025.1750002

**Published:** 2026-06-08

**Authors:** Elif Yaprak Çolak, Nizami Duran

**Affiliations:** Department of Medical Microbiology, Medical Faculty, Hatay Mustafa Kemal University, Antakya-Hatay, Türkiye

**Keywords:** postbiotics, linezolid, amikacin, nosocomial infections, antimicrobial synergy, microbiome therapy

In the published article, several references were incorrectly written due to bibliographic and typographical errors. The references have now been corrected as detailed below. These corrections concern only the accuracy of the reference list and citation formatting. No changes have been made to the scientific content, results, interpretation, or conclusions of the study.

The reference “Abed et al., 2021” was incorrectly written. It should be:

“Jayashree S., Karthikeyan R., Nithyalakshmi S., Ranjani J., Gunasekaran P., Rajendhran J. Anti-adhesion Property of the Potential Probiotic Strain Lactobacillus fermentum 8711 Against Methicillin-Resistant Staphylococcus aureus (MRSA). Front Microbiol. 2018 Mar 8; 9:411. doi: 10.3389/fmicb.2018.00411”.

The reference “Aguilar-ToaláJ. E. et al., 2018” was incorrectly written. It should be:

Aguilar-Toalá J.E., Garcia-Varela R., Garcia H.S., Mata-Haro V., González-Córdova A.F., Vallejo-Cordoba B., et al. Postbiotics: An evolving term within the functional foods field. Trends Food Sci Technol. 2018; 75:105–114. doi: 10.1016/j.tifs.2018.03.009.

The reference “Asadi A., Akrami S., Hajipour N., 2024” was incorrect. The correct citation is:

“Asadi Z., Abbasi A., Ghaemi A., Montazeri E.A., Akrami S. Investigating the properties and antibacterial, antioxidant, and cytotoxicity activity of postbiotics derived from Lacticaseibacillus casei on various gastrointestinal pathogens *in vitro* and in food models. GMS Hyg Infect Control. 2024;19:Doc60. doi: 10.3205/dgkh000515”.

The reference “Da C. et al., 2025” was incorrect. It should be:

“Calvanese C.M., Villani F., Ercolini D., De Filippis F. Postbiotics versus probiotics: Possible new allies for human health. Food Res Int. 2025;217:116869. doi: 10.1016/j.foodres.2025.116869”.

The reference “Doe J., Smith A., Kumar R., 2025” was incorrectly written. The correct citation is:

“Pristavu M.C., Diguță F.C., Aldea A.C., Badea F., Dragoi Cudalbeanu M., Ortan A., Matei F. Functional Profiling of Enterococcus and Pediococcus Strains: An *In Vitro* Study on Probiotic and Postbiotic Properties. Microorganisms. 2025;13(6):1348. doi: 10.3390/microorganisms13061348“.

The reference “Garg A. et al., 2024” was incorrect. The correct citation is:

“Aghebati-Maleki L., Hasannezhad P., Abbasi A., Khani N. Antibacterial, Antiviral, Antioxidant, and Anticancer Activities of Postbiotics: A Review of Mechanisms and Therapeutic Perspectives. Biointerface Res Appl Chem. 2022;12(2):2629–2645. doi: 10.33263/BRIAC122.26292645”.

The reference “Matsuoka Y. et al., 2020” was incorrect. The correct citation is:

“Zipperer A., Scheurer J., Kretschmer D. Cytotoxicity Assays as Predictors of the Safety and Efficacy of Antimicrobial Agents. Methods Mol Biol. 2023;2601:153–167. doi: 10.1007/978-1-0716-2855-3_8”.

The reference “Ribeiro S.M. et al., 2024” was incorrect. The correct citation is:

“Taheri-Araghi S. Synergistic Action of Antimicrobial Peptides and Antibiotics: Current Understanding and Future Directions. Front Microbiol. 2024;15:1390765. doi: 10.3389/fmicb.2024.1390765”.

The reference “Smith A. et al., 2025” was incorrect. The correct citation is:

“Meena K.K., Joshi M., Gupta L., Meena S. Comprehensive Insights into Postbiotics: Bridging the Gap to Real-World Application. Food Nutr. 2025;1:100024. doi: 10.1016/j.fnutr.2025.100024”.

The reference “Ünal E.B. et al., 2024” was incorrectly written. The correct citation is:

“García-Gamboa R., Perfecto-Avalos Y., Gonzalez-Garcia J., et al. *In vitro* analysis of postbiotic antimicrobial activity against Candida species in a minimal synthetic model simulating the gut mycobiota in obesity. Sci Rep. 2024;14:16760. doi: 10.1038/s41598-024-66806-3”.

The reference “Zhang X. et al., 2025” was incorrect. The correct citation is:

“Jahedi S., Pashangeh S. Bioactivities of Postbiotics in Food Applications: A Review. Iran J Microbiol. 2025;17(3):348–357. doi: 10.18502/ijm.v17i3.18816”.

The reference “Zhang Y. et al., 2024” was incorrectly written. The correct citation is:

“Zdybel K., Śliwka A., Polak-Berecka M., Polak P., Waśko A. Postbiotics Formulation and Therapeutic Effect in Inflammation: A Systematic Review. Nutrients. 2025;17:2187. doi: 10.3390/nu17132187”.

The original version of this article has been updated.

