# Supplementary material for: Synergistic antibacterial effects of postbiotics combined with linezolid and amikacin against nosocomial pathogens
Source: Front Cell Infect Microbiol. 2025 Aug 14;15:1616501. doi: 10.3389/fcimb.2025.1616501 (PMC12390976; doi:10.3389/fcimb.2025.1616501)
Supplement: Supplementary Figure 1 — Workflow of the methodology applied in this study, including postbiotic extraction, quantification (Bradford Assay), antimicrobial combination testing (binary and ternary mixtures), and cytotoxicity assay (MTT-based evaluation on Vero cells). [file Table1.docx]

**Supplementary Figure S1**: Experimental workflow of postbiotic extraction, quantification, antimicrobial synergy testing, and cytotoxicity assay.


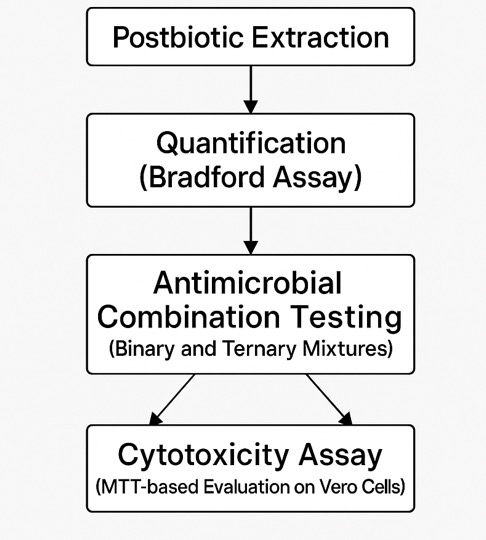


**Figure 17.** Comparative evaluation of the efficacy against *S. aureus* of the combination of *S. thermophilus* and *L. casei* postbiotics, linezolid treatment alone, and the combination of amikacin with postbiotics.

| Tukey's multiple comparisons test | Summary | P Value |
| --- | --- | --- |
| 1^st^hour |  |  |
| Postbiotics of ST+LC vs. Linezolid | ns | >0,9999 |
| Postbiotics of ST+LC vs. Linezolid+Postbiotics of ST+LC | **** | <0,0001 |
| Linezolid vs. Linezolid+Postbiotics of ST+LC | **** | <0,0001 |
| 2^nd^hour |  |  |
| Postbiotics of ST+LC vs. Linezolid | **** | <0,0001 |
| Postbiotics of ST+LC vs. Linezolid+Postbiotics of ST+LC | **** | <0,0001 |
| Linezolid vs. Linezolid+Postbiotics of ST+LC | **** | <0,0001 |
| 3^rd^hour |  |  |
| Postbiotics of ST+LC vs. Linezolid | *** | 0,0002 |
| Postbiotics of ST+LC vs. Linezolid+Postbiotics of ST+LC | **** | <0,0001 |
| Linezolid vs. Linezolid+Postbiotics of ST+LC | ** | 0,0020 |
| 4^th^hour |  |  |
| Postbiotics of ST+LC vs. Linezolid | * | 0,0216 |
| Postbiotics of ST+LC vs. Linezolid+Postbiotics of ST+LC | **** | <0,0001 |
| Linezolid vs. Linezolid+Postbiotics of ST+LC | ** | 0,0020 |

**Figure 18.** Comparative evaluation of the efficacy against *S. aureus* of the combination of *L.casei* and *L. bulgaricus* postbiotics, linezolid treatment alone, and the combination of amikacin with postbiotics.

| Tukey's multiple comparisons test | Summary | P Value |
| --- | --- | --- |
| 1^st^hour |  |  |
| Postbiotics of ST+LC vs. Linezolid | ns | >0,9999 |
| Postbiotics of ST+LC vs. Linezolid+Postbiotics of ST+LC | **** | <0,0001 |
| Linezolid vs. Linezolid+Postbiotics of ST+LC | **** | <0,0001 |
| 2^nd^hour |  |  |
| Postbiotics of ST+LC vs. Linezolid | **** | <0,0001 |
| Postbiotics of ST+LC vs. Linezolid+Postbiotics of ST+LC | **** | <0,0001 |
| Linezolid vs. Linezolid+Postbiotics of ST+LC | **** | <0,0001 |
| 3^rd^hour |  |  |
| Postbiotics of ST+LC vs. Linezolid | *** | 0,0002 |
| Postbiotics of ST+LC vs. Linezolid+Postbiotics of ST+LC | **** | <0,0001 |
| Linezolid vs. Linezolid+Postbiotics of ST+LC | ** | 0,0020 |
| 4^th^hour |  |  |
| Postbiotics of ST+LC vs. Linezolid | * | 0,0216 |
| Postbiotics of ST+LC vs. Linezolid+Postbiotics of ST+LC | **** | <0,0001 |
| Linezolid vs. Linezolid+Postbiotics of ST+LC | ** | 0,0020 |

**Figure 19.** Comparative evaluation of the efficacy against *E.coli* of the combination of *S.thermophilus* and *L. bulgaricus* postbiotics, amikacin treatment alone, and the combination of amikacin with postbiotics.

| Tukey's multiple comparisons test | Summary | P Value |
| --- | --- | --- |
| 1^st^hour |  |  |
| Postbiotics of ST+LC vs. Amikacin | ns | 0,5091 |
| Postbiotics of ST+LC vs. Amikacin+Postbiotics of ST+LC | **** | <0,0001 |
| Amikacin vs. Amikacin+Postbiotics of ST+LC | **** | <0,0001 |
| 2^nd^hour |  |  |
| Postbiotics of ST+LC vs. Amikacin | ** | 0,0031 |
| Postbiotics of ST+LC vs. Amikacin+Postbiotics of ST+LC | **** | <0,0001 |
| Amikacin vs. Amikacin+Postbiotics of ST+LC | **** | <0,0001 |
| 3^rd^hour |  |  |
| Postbiotics of ST+LC vs. Amikacin | * | 0,0424 |
| Postbiotics of ST+LC vs. Amikacin+Postbiotics of ST+LC | **** | <0,0001 |
| Amikacin vs. Amikacin+Postbiotics of ST+LC | *** | 0,0003 |
| 4^th^hour |  |  |
| Postbiotics of ST+LC vs. Amikacin | * | 0,0339 |
| Postbiotics of ST+LC vs. Amikacin+Postbiotics of ST+LC | **** | <0,0001 |
| Amikacin vs. Amikacin+Postbiotics of ST+LC | ** | 0,0070 |

**Figure 20.** Comparative evaluation of the efficacy against *E.coli* of the combination of *L.casei* and *L. bulgaricus* postbiotics, amikacin treatment alone, and the combination of amikacin with postbiotics.

| Tukey's multiple comparisons test | Summary | P Value |
| --- | --- | --- |
| 1^st^hour |  |  |
| Postbiotics of LC+LB vs. Amikacin | ns | 0,9290 |
| Postbiotics of LC+LB vs. Amikacin+Postbiotics of LC+LB | **** | <0,0001 |
| Amikacin vs. Amikacin+Postbiotics of LC+LB | **** | <0,0001 |
| 2^nd^hour |  |  |
| Postbiotics of LC+LB vs. Amikacin | * | 0,0177 |
| Postbiotics of LC+LB vs. Amikacin+Postbiotics of LC+LB | **** | <0,0001 |
| Amikacin vs. Amikacin+Postbiotics of LC+LB | **** | <0,0001 |
| 3^rd^hour |  |  |
| Postbiotics of LC+LB vs. Amikacin | ns | 0,0870 |
| Postbiotics of LC+LB vs. Amikacin+Postbiotics of LC+LB | **** | <0,0001 |
| Amikacin vs. Amikacin+Postbiotics of LC+LB | **** | <0,0001 |
| 4^th^hour |  |  |
| Postbiotics of LC+LB vs. Amikacin | ns | 0,7980 |
| Postbiotics of LC+LB vs. Amikacin+Postbiotics of LC+LB | * | 0,0126 |
| Amikacin vs. Amikacin+Postbiotics of LC+LB | ns | 0,0526 |

**Figure 21.** Comparative evaluation of the efficacy against *P.aeruginosa* of *S.thermophilus* and *L. casei* postbiotics, amikacin treatment alone, and the combination of amikacin with postbiotics.

| Tukey's multiple comparisons test | Summary | P Value |
| --- | --- | --- |
|  |  |  |
| 1^st^hour |  |  |
| Postbiotics of ST+LC vs. Amikacin | ** | 0,0095 |
| Postbiotics of ST+LC vs. Amikacin+Postbiotics of ST+LC | **** | <0,0001 |
| Amikacin vs. Amikacin+Postbiotics of ST+LC | ** | 0,0011 |
|  |  |  |
| 2^nd^hour |  |  |
| Postbiotics of ST+LC vs. Amikacin | *** | 0,0009 |
| Postbiotics of ST+LC vs. Amikacin+Postbiotics of ST+LC | **** | <0,0001 |
| Amikacin vs. Amikacin+Postbiotics of ST+LC | **** | <0,0001 |
|  |  |  |
| 3^rd^hour |  |  |
| Postbiotics of ST+LC vs. Amikacin | **** | <0,0001 |
| Postbiotics of ST+LC vs. Amikacin+Postbiotics of ST+LC | **** | <0,0001 |
| Amikacin vs. Amikacin+Postbiotics of ST+LC | * | 0,0107 |
|  |  |  |
| 4^th^hour |  |  |
| Postbiotics of ST+LC vs. Amikacin | *** | 0,0008 |
| Postbiotics of ST+LC vs. Amikacin+Postbiotics of ST+LC | **** | <0,0001 |
| Amikacin vs. Amikacin+Postbiotics of ST+LC | * | 0,0208 |

**Figure 22.** Comparative evaluation of the efficacy against *P.aeruginosa* of *L. casei* and *L.bulgaricus* postbiotics, amikacin treatment alone, and the combination of amikacin with postbiotics.

| Tukey's multiple comparisons test | Summary | P Value |
| --- | --- | --- |
|  |  |  |
| 1^st^hour |  |  |
| Postbiotics of LC+LB vs. Amikacin | **** | <0,0001 |
| Postbiotics of LC+LB vs. Amikacin+Postbiotics of LC+LB | **** | <0,0001 |
| Amikacin vs. Amikacin+Postbiotics of LC+LB | **** | <0,0001 |
|  |  |  |
| 2^nd^hour |  |  |
| Postbiotics of LC+LB vs. Amikacin | **** | <0,0001 |
| Postbiotics of LC+LB vs. Amikacin+Postbiotics of LC+LB | **** | <0,0001 |
| Amikacin vs. Amikacin+Postbiotics of LC+LB | **** | <0,0001 |
|  |  |  |
| 3^rd^hour |  |  |
| Postbiotics of LC+LB vs. Amikacin | **** | <0,0001 |
| Postbiotics of LC+LB vs. Amikacin+Postbiotics of LC+LB | **** | <0,0001 |
| Amikacin vs. Amikacin+Postbiotics of LC+LB | **** | <0,0001 |
|  |  |  |
| 4^th^hour |  |  |
| Postbiotics of LC+LB vs. Amikacin | **** | <0,0001 |
| Postbiotics of LC+LB vs. Amikacin+Postbiotics of LC+LB | **** | <0,0001 |
| Amikacin vs. Amikacin+Postbiotics of LC+LB | **** | <0,0001 |

**Figure 23.** Comparative evaluation of the efficacy against *P.mirabilis* of *S.thermophilus* and *L. casei* postbiotics, amikacin treatment alone, and the combination of amikacin with postbiotics.

| Tukey's multiple comparisons test | Summary | P Value |
| --- | --- | --- |
| 1^st^hour |  |  |
| Postbiotics of ST+LC vs. Amikacin | * | 0,0106 |
| Postbiotics of ST+LC vs. Amikacin+Postbiotics of ST+LC | **** | <0,0001 |
| Amikacin vs. Amikacin+Postbiotics of ST+LC | *** | 0,0002 |
| 2^nd^hour |  |  |
| Postbiotics of ST+LC vs. Amikacin | ** | 0,0055 |
| Postbiotics of ST+LC vs. Amikacin+Postbiotics of ST+LC | **** | <0,0001 |
| Amikacin vs. Amikacin+Postbiotics of ST+LC | **** | <0,0001 |
| 3^rd^hour |  |  |
| Postbiotics of ST+LC vs. Amikacin | **** | <0,0001 |
| Postbiotics of ST+LC vs. Amikacin+Postbiotics of ST+LC | **** | <0,0001 |
| Amikacin vs. Amikacin+Postbiotics of ST+LC | *** | 0,0003 |
| 4^th^hour |  |  |
| Postbiotics of ST+LC vs. Amikacin | **** | <0,0001 |
| Postbiotics of ST+LC vs. Amikacin+Postbiotics of ST+LC | **** | <0,0001 |
| Amikacin vs. Amikacin+Postbiotics of ST+LC | * | 0,0110 |

**Figure 24.** Comparative evaluation of the efficacy against *P.mirabilis* of *L. casei* and *L.bulgaricus* postbiotics, amikacin treatment alone, and the combination of amikacin with postbiotics.

| Tukey's multiple comparisons test | Summary | P Value |
| --- | --- | --- |
|  |  |  |
| 1^st^hour |  |  |
| Postbiotics of LC+LB vs. Amikacin | * | 0,0146 |
| Postbiotics of LC+LB vs. Amikacin+Postbiotics of LC+LB | **** | <0,0001 |
| Amikacin vs. Amikacin+Postbiotics of LC+LB | **** | <0,0001 |
|  |  |  |
| 2^nd^hour |  |  |
| Postbiotics of LC+LB vs. Amikacin | **** | <0,0001 |
| Postbiotics of LC+LB vs. Amikacin+Postbiotics of LC+LB | **** | <0,0001 |
| Amikacin vs. Amikacin+Postbiotics of LC+LB | ** | 0,0078 |
|  |  |  |
| 3^rd^hour |  |  |
| Postbiotics of LC+LB vs. Amikacin | **** | <0,0001 |
| Postbiotics of LC+LB vs. Amikacin+Postbiotics of LC+LB | **** | <0,0001 |
| Amikacin vs. Amikacin+Postbiotics of LC+LB | ** | 0,0078 |
|  |  |  |
| 4^th^hour |  |  |
| Postbiotics of LC+LB vs. Amikacin | **** | <0,0001 |
| Postbiotics of LC+LB vs. Amikacin+Postbiotics of LC+LB | **** | <0,0001 |
| Amikacin vs. Amikacin+Postbiotics of LC+LB | ** | 0,0086 |
